# Stable, Efficient, Copper Coordination Polymer-Derived Heterostructured Catalyst for Oxygen Evolution under pH-Universal Conditions

**DOI:** 10.1021/acsami.1c01424

**Published:** 2021-05-21

**Authors:** Ligang Wang, Ning Ma, Nian Wu, Xiaoge Wang, Junjie Xin, Dingsheng Wang, Jianhua Lin, Xingguo Li, Junliang Sun

**Affiliations:** † College of Chemistry and Molecular Engineering, and Beijing National Laboratory for Molecular Sciences (BNLMS), Peking University, 5 Yiheyuan Road, Beijing 100871, P. R. China; ‡ Hubei Key Laboratory of Polymer Materials, Key Laboratory for the Green Preparation and Application of Functional Materials (Ministry of Education), School of Materials Science and Engineering, Hubei University, Wuhan 430062, P. R. China; § Institute for Interdisciplinary Information Sciences, Tsinghua University, Beijing 100084, P. R. China; ∥ Department of Chemistry, Tsinghua University, Beijing 100084, China

**Keywords:** interfacial effect, Cu-based coordination polymer, OER, water splitting, wide pH values

## Abstract

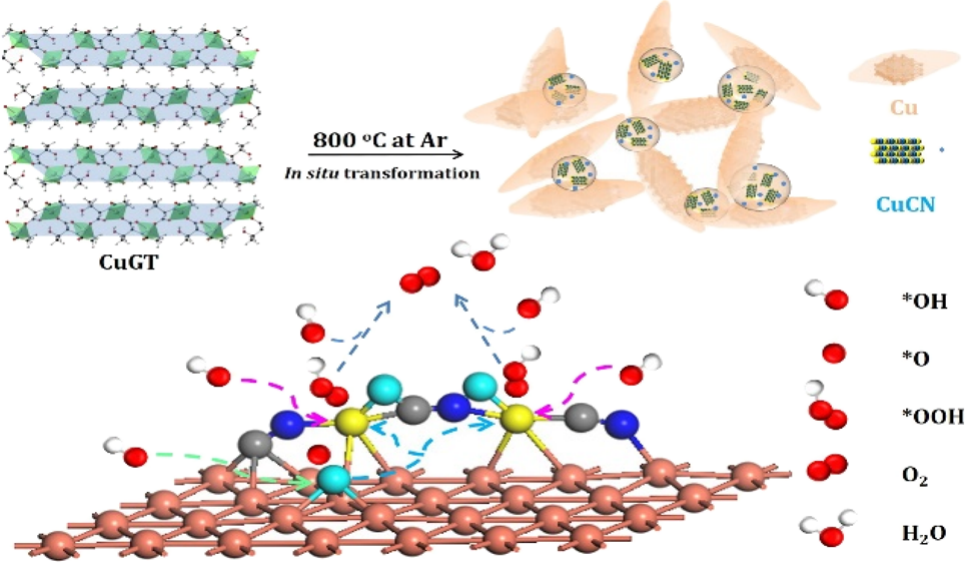

The constructure of a heterostructured interface is an effective way to design highly durable and efficient water oxidation electrocatalysts. Herein, Cu/CuCN with heterointerfaces is the first synthesized case through a simple epitaxial-like growth method, displaying superior activity and stability under pH-universal media. Associated with high electron transport and transfer of the epitaxial interfacial area, the Cu/CuCN pre-catalyst is applied to deliver the oxygen evolution reaction (OER) with lower overpotentials of 250 mV (forward scan) and 380 mV (backward scan) at 10 mA cm^–2^ and demonstrates better intrinsic activity (*j*_ECSA_ of 1.0 mA cm^–2^ at 420 mV) and impressive stability (136 h) in 1.0 M KOH, which exceeds most previous catalysts. Even using a nominal voltage of 1.5 V of a AA battery can drive the overall water-splitting setup. Experiments combined with theoretical simulations further uncover the existence of CuO species at the heterointerface during basic OER, which is evidence of better OER performance with abundant active sites that accelerate the conversion kinetics.

## Introduction

Currently, hydrogen (H_2_) due to its high energy density, renewability, and ecofriendly traits is believed to be a good choice to replace fossil fuels to address energy and environmental issues.^1,2^ In general, electrochemically driven water splitting into H_2_ and O_2_ is an emerging strategy based on the occurrence of two main reactions: the hydrogen and oxygen production reaction. It is common knowledge that it is the anode oxygen evolution reaction (OER) that mainly limits the efficiency of water splitting because of its slow dynamics where water is oxidized to O_2_.^3^ At the same time, noble metal-based oxides (such as Ir and Ru) are usually used as excellent OER catalysts; however, their scarce reserves and cost problems cause some difficulties in large-scale commercial applications.^4^ With the development of catalysts such as metal oxides, phosphates, and nitrides, the OER catalysts have been widely used for clean energy conversion technology. However, excellent catalysts with high conductivity to facilitate charge transfer, abundant active sites, better long-term stability, and low prices as well as possessing a high surface area are still very scarce and are greatly desired in water electrolysis technology. So far, several strategies have been explored for achieving exceptional OER electrochemical performance, including nanoscopic confinement,^5^ core–shell structuring,^6^ defect engineering,^7,8^ strain engineering,^9,10^ corrosion engineering,^11^ synergistic active site modulation,^12,13^ doping,^14−17^ and so on.

Predictably, surface/interface heterostructure engineering is yet to be a promising effective strategy to optimize electrocatalytic activity with unique properties, which is mainly attributed to the surface structure and interface interactions for the catalyst has a vital effect on the activity and the potential gradient between the interfaces can powerfully boost charge separation and migration.^18−23^ It is well accepted that tuning the surface atom environment and the interface binding strength can largely improve the catalytic activity, such as promoting charge transfer kinetics and increasing the number or enhancing the intrinsic activity of active sites. Notably, a potential measure is to advisably grow epitaxial heterostructure interfaces with rich phase boundaries, which are attractive for an electrochemical application of surface regulation.^24−26^ In addition, the synergistic effect originating from the existence of heterogeneous interfaces also provides the possibility of modulating their electronic structure and appearing with distinctive atomic coordination. Considering the separate composition for a specific catalytic reaction, heterostructure engineering will play a more significant synergistic role.

Metal–organic frameworks (MOFs), with ultrahigh specific surface area structures and rich metal active sites, have been widely studied as ideal candidate materials for electrocatalysis during the last few decades.^27−30^ Furthermore, FeCoNi-based MOF derivatives, for instance, metals and metal oxides,^31−33^ sulfides,^34,35^ and phosphides,^36−38^ by calcining in different atmospheres, are usually more stable and have widespread applications than MOFs themselves for long-term OER. However, a few examples have committed to Cu-MOFs or their derivatives were used for OER catalysts. Considering that the excellent conductivity to accelerate interfacial electron transport and reaction kinetics, higher abundance, and lower price from Cu-based MOFs, compared with cobalt and nickel, which is very essential for designing efficient catalysts. Therefore, the development of novel Cu-based MOF electrocatalysts for OER is highly desirable, especially heterointerface OER catalysts. Recently, a few other groups have also reported that Cu@CuO–C or CuO from Cu-EA is an active catalyst for OER.^39,40^ Unfortunately, based on the incompatibility of the electrocatalyst in different pH values, it cannot achieve OER activity in the same electrolyte.^41^ However, considering the actual needs for water electrolysis technology, it is vital to develop highly efficient and durable heterostructured copper-based OER electrocatalysts that work well in pH-universal electrolyte conditions.

Herein, we propose an *in situ* growth strategy to increase the activity of Cu-based quasi-MOF materials by forming a copper core-supported Cu–C/N (named Cu/CuCN) heterostructure to activate the basal planes of the Cu species with better dispersed CuCN active sites. In detail, Cu/CuCN is synthesized through calcining a two-dimensional Cu–peptide coordination polymer, which can obtain carbon and nitrogen from raw materials (see the Experimental Section). Notably, heterostructured Cu/CuCN electrocatalysts, for the first time, were used as OER catalysts with superior catalytic activity: low Tafel values of 76, 98, and 92 mV dec^–1^ in basic, acidic, and neutral electrolyzer systems, respectively. Furthermore, experimental and theoretical calculations confirmed the existence of rich CuO active species during basic OER. We concluded that the active sites of epitaxial interfaces can significantly accelerate OER. The reasonable explanations are that the kinetic barrier for water molecule activation is decreased, the transformation efficiency of *OH to *O intermediates on the active CuO surface is improved, and the transformation efficiency of *OH to *OOH/*O_2_ intermediates on active Cu sites from oxygen-embedded CuCN is enhanced. Moreover, the needed voltage of only 1.53 V can drive a 10 mA cm^–2^ current density for a two-electrode system. This superior heterostructure system presents unprecedented OER catalytic activity and stability at a relatively low overpotential in wide pH-range environments.

## Experimental Section

### Materials and Chemicals

NaOH was purchased from Shanghai Reagent Chemical Co. (China). GT was purchased from Shanghai Apeptide Co. Ltd. (China). Glutamic acid and dicyandiamide were purchased from Aladdin Reagent Co. Ltd. (China). Cu(NO_3_)_2_·3H_2_O was purchased from Xilong Chemical Co., Ltd. H_2_SO_4_ solution and KOH were purchased from Sigma-Aldrich. NaH_2_PO_4_·2H_2_O and Na_2_HPO_4_·12H_2_O were purchased from Beijing Chemical Plant. Commercial carbon paper was obtained from Nanjing MKT Co., Ltd. Commercial copper(I) cyanide was purchased from J&K Scientific Ltd.

### Synthesis of CuGT

Cu-(Gly-Thr) (CuGT; Gly-Thr = C_6_H_12_N_2_O_4_) was prepared as in a previous study.^42^ GT (11 mg, 0.0625 mmol) and 8 mg (0.0331 mmol) of Cu(NO_3_)_2_·3H_2_O were added into a 4 mL scintillation vial containing 3 mL of methanol. The mixture was sonicated for 10 min followed by addition of 80 μL of 1 M NaOH (aq). Next, the blue methanolic solution was shaken on a vortex mixer for 10 min, giving rise to the formation of blue crystals suitable for X-ray crystallography (63% yield). The crystalline product was then filtered from the reaction mixture, thoroughly washed with CH_3_OH, and sealed. The blue powder was dried in an oven at 100 °C for 1 h.

### Synthesis of Cu/CuCN and Cu NPs

A grinding method was used to mix CuGT (0.1 g) with dicyandiamide (0.3 g). The mixture was calcined at 800 °C for 3 h in an Ar atmosphere with an increasing rate of 2 °C min^–1^, denoted as Cu/CuCN. At the same time, the obtained catalysts, named Cu NPs, were only from CuGT after 800 °C calcination. As controls, we adjusted the various ratios of CuGT to D (dicyandiamide) of 1:1, 1:3, 1:10, 1:50, and 1:100 during the catalyst synthesis stage. Among these, the obtained catalysts were named Cu–CuCN from the ratios of CuGT to D (1:1). In addition, the prepared samples after calcination of CuGT and CuGT + D at 700 °C were denoted as Cu NPs-1 and Cu/CuCN-1, respectively. Similarly, the prepared samples after calcination of CuGT and CuGT + D at 600 °C were denoted as Cu NPs-2 and Cu/CuCN-2, respectively.

### Synthesis of Cu(NO_3_)_2_-Based Catalyst

Cu(NO_3_)_2_·3H_2_O (0.5 g), dicyandiamide (D, 1.0 g), and glutamic acid (Glu, 0.5 g) were mixed together to obtain the precursor. Subsequently, the mixture was calcined at different temperatures (such as 600/700/800 °C) under the same conditions as Cu/CuCN, denoted as Cu(NO_3_)_2_ + Glu + D. In addition, the Cu(NO_3_)_2_ + Glu, Cu(NO_3_)_2_ + D, and Cu(NO_3_)_2_ catalysts were also prepared using the same method mentioned from the 0.5 g of Cu(NO_3_)_2_·3H_2_O with 0.5 g of Glu, 0.5 g of Cu(NO_3_)_2_·3H_2_O with 1.0 g of D, and only 0.5 g of Cu(NO_3_)_2_·3H_2_O, respectively.

### Synthesis of MoNi_4_/MoO_3–*x*_ Catalyst

We synthesized the MoNi_4_/MoO_3–*x*_ catalyst by methods previously reported by Hu’s group.^43^ In brief, treated nickel foam was submerged in a solution containing 30 mL of (NH_4_)_6_Mo_7_O_24_·4H_2_O and NH_4_F at 150 °C for 8 h in an oven and then cooled down to room temperature. Lastly, the obtained NiMoO_4_ precursor was used for pyrolysis at 350 °C for 1.5 h in H_2_/Ar to obtain the MoNi_4_/MoO_3–*x*_ catalyst.

### Characterizations

The morphology of the prepared sample was analyzed by field-emission scanning electron microscopy (FESEM, Hitachi S-4800 and Zeiss Merlin compact) and field-emission transmission electronic microscopy (FETEM) (JEOL-2100F, JEOL Ltd., Japan). Elemental distribution maps were collected by energy-dispersive spectrometry (EDS, Bruker Xflash 6100) using an accelerating voltage of 15 kV. Powder X-ray diffraction (PXRD) was performed using an X-Pert3 powder (PANalytical, the Netherlands) diffractometer with Cu Kα1 (λ = 1.5406 Å) radiation. X-ray photoelectron spectroscopy (XPS, Al Kα radiation, and *h*ν = 1486.6 eV) was performed to reveal the chemical compositions and valence state using the C 1s peak of the C–C and C–H bonds located at 284.8 eV as reference. The CasaXPS software was adapted to conduct the peak fitting. Thermogravimetric–mass spectrometric (TG-MS) analysis was carried out using an STA449C/Qms 403C. The Raman spectra of the prepared catalysts were collected with Jobin Yvon-Horiba LabRam ARAMIS systems with 532 nm excitation lasers. A Micromeritics ASAP2020 device (at 77 K) was employed to perform the N_2_ adsorption–desorption test. Infrared (IR) spectra were collected using a Fourier transform infrared spectrometer (Nicolet is50, ThermoFisher Co.).

### Working Electrode Preparation

First, 2.0 mg of the obtained Cu/CuCN catalyst was mixed with 500 μL of isopropyl alcohol (IPA, Sigma-Aldrich) and 20 μL of Nafion (5 wt %, Sigma-Aldrich). Then, 10 μL of the prepared solution was spun onto carbon paper (CP). Finally, the area of the working electrode is around 0.5 cm^2^. Therefore, the catalyst loading was about 76.9 μg_metal_ cm^–2^.

### Electrochemical Measurements

Electrochemical tests were performed using CHI 760E equipment at room temperature in various electrolytes (1.0 M KOH, 0.5 M H_2_SO_4_, and 0.1 M phosphate-buffered solution (PBS, pH 7.0)) with N_2_-saturated solutions. A three-electrode system was used in the experiment, and a graphite rod and Hg/HgO (in basic) or Ag/AgCl filled with saturated KCl (in acidic) were employed as the counter electrode and reference electrode, respectively. The electrochemical tests were recorded with a 5 mV s^–1^ scan rate, and all data were collected without the *iR* correction. The electrochemical impedance spectrum (EIS) was collected at 1.53 V (vs RHE) with a 10 kHz to 1 Hz frequency range. The electrochemically active surface area (ECSA) was obtained by dealing with the relationship between current density and scan rates in pH-universal conditions. A linear relationship was obtained by plotting the difference of current densities (*J*) with the change in scan rate between the anodic and cathodic sweeps (*J*_anodic_ – *J*_cathodic_) at fixed voltage. The value of the geometric double layer capacitance (*C*_dl_) can be obtained by fitting the line slope. The ECSA of a catalyst on CP is estimated according to the equation ECSA = *C*_dl_/*C*_s_, where *C*_s_ is the specific capacitance value of an ideal flat surface with a 1 cm^–2^ real surface area. Generally, the value of 60 μF cm^–2^ is used for the description of *C*_s_.^44^ The catalytic durability test was performed at a constant current density.

### Theoretical Calculations

Density functional theory (DFT) calculations were carried out using the Vienna ab initio simulation package (VASP).^45^ In all calculations, we have considered spin polarization. The exchange–correlation was achieved by the generalized gradient approximation (GGA) described by Perdew, Burke, and Ernzerhof (PBE).^46^ A 3 × 3 × 1 Monkhorst–Pack *k* point grid for optimization and a 5 × 5 × 1 grid for the density of states were used to describe the Brillouin zone.^47^ A smearing parameter of 0.1 eV was adapted for Gaussian smearing. For the model of the CuCN-embedded Cu(111) facet, four layers of Cu atoms and 28 atoms per layer were used as the Cu(111) model with a vacuum layer of over 14 Å, above which three repeated units of CuCN extracted from crystal CuCN were horizontally sited as the preliminary configuration for optimization. O atoms were initially put at various sites near CuCN to obtain stabilized configurations. In the process of geometry optimization, the conjugate gradient method was employed. For details, the two atomic layers at the bottom were in a relaxed state until the Hellmann–Feynman force was smaller than 0.01 eV/Å on each atom.

## Results and Discussion

### Synthesis and Characterization of Fusiform Cu/CuCN

**Figure 1:**
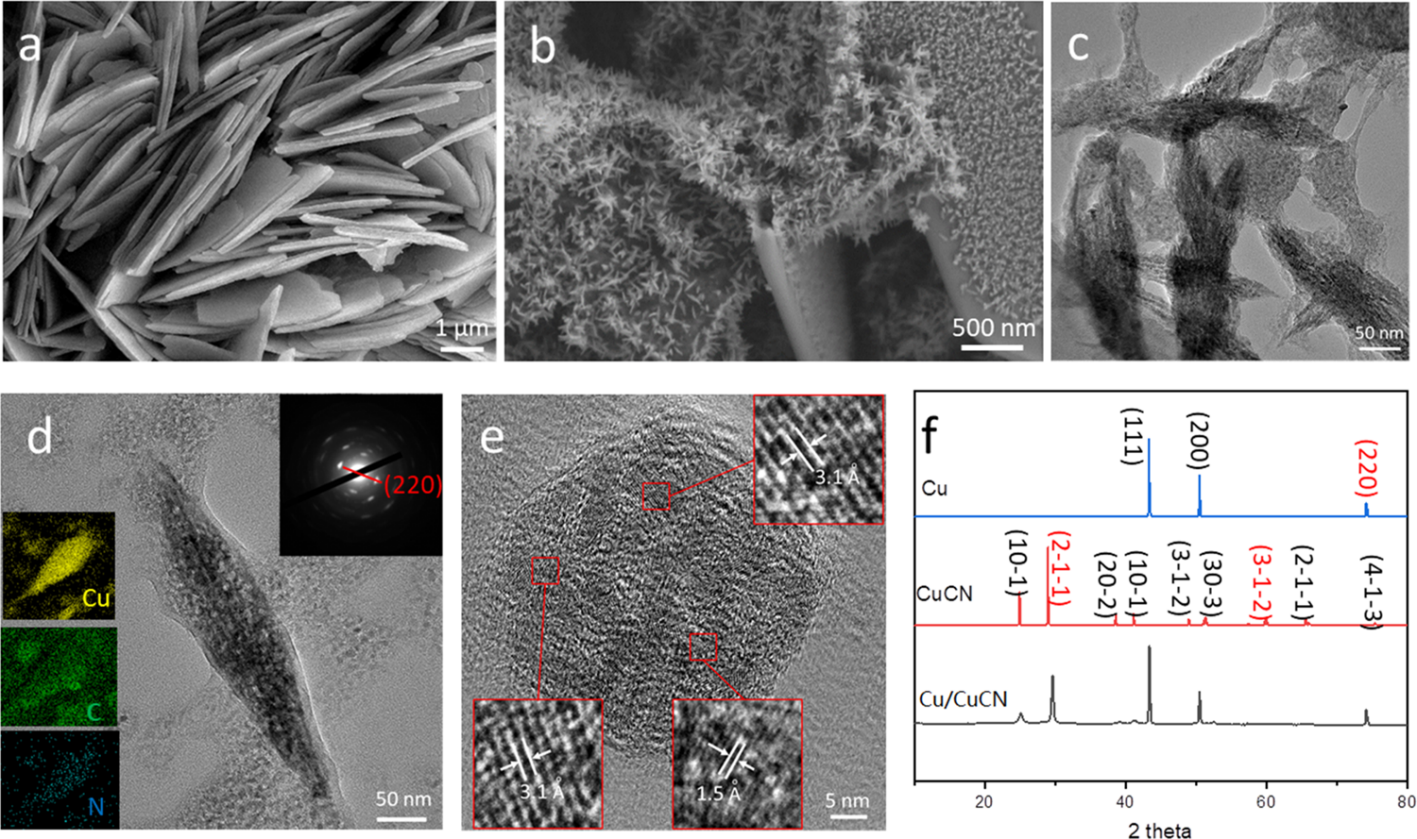
Characterization of Cu/CuCN heterostructure electrodes for OER. (a) SEM image of the CuGT. (b) SEM image of Cu/CuCN-1. (c, d) TEM image of Cu/CuCN and insets of panel (d): corresponding SAED pattern and EDS elemental mapping of copper, carbon, and nitrogen. (e) HRTEM image of Cu/CuCN. (f) PXRD patterns of the simulated Cu, simulated CuCN, and obtained Cu/CuCN samples.

**Scheme 1:**
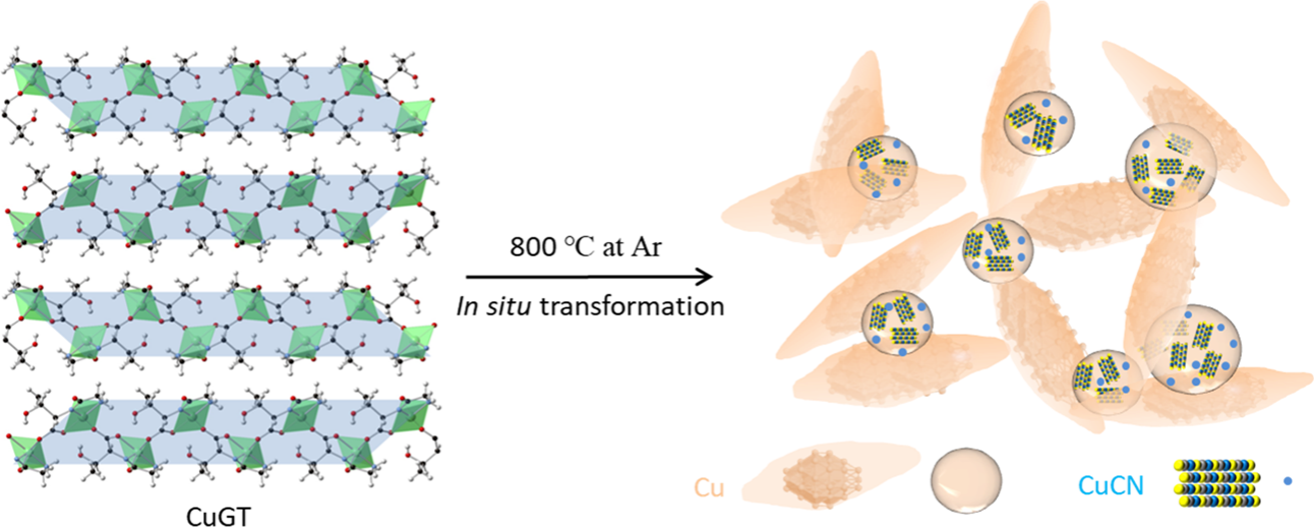
Illustration of the Synthesis Method and Obtained Structure of Cu/CuCN

The overall fabrication process of the spindle-shaped Cu/CuCN is shown in Scheme 1. First, a copper–peptide coordination MOF (CuGT)^42^ served as the source of Cu precursors and a support material (Figure S1). This process ultimately results in the formation of novel Cu/CuCN nanocomposite materials (see the details in the Experimental Section). Field-emission scanning electron microscope (FESEM) images present that CuGT is predominantly layered in shape with an average thickness of 100 nm, as shown in Figure 1a. Then, Cu/CuCN was obtained by direct thermal treatment at 800 °C in an argon atmosphere. As a control, we adjusted the ratio of CuGT to dihydrodiamine and compared the influences of various temperatures on the synthesis of catalysts (Figures S2–S9). As shown in the typical SEM image of as-synthesized Cu/CuCN (Figure S10), such materials adopted a gossamer shape decorated with small particles at the quantum dot level. Moreover, Figure 1b also clearly shows the SEM image of Cu/CuCN-1 obtained at 700 °C in an argon atmosphere. Meanwhile, the results of SEM and powder X-ray diffraction (PXRD) from Cu(NO_3_)_2_ as a copper source precursor indicated that almost no highly active CuCN is present in the catalyst (Figures S11–S16).

After preparation by the ultrasonic dispersion method, the TEM images display that the average size of spindle-shaped Cu/CuCN is around 350 nm long and 100 nm wide, as shown in Figure 1c,d. The catalyst’s detailed morphology was investigated by high-resolution TEM (HRTEM) coupled selected-area electron diffraction (SAED) analyses, as shown in Figure 1d,e. Based on the SAED analyses, the *d* value of the main section of the nanoshuttle is 1.28 Å, corresponding to the (220) crystal plane of Cu (Figure 1d). The HRTEM image illustrates that the interplanar distances of the sub-nanocrystal in red boxes are measured to be 3.1 and 1.5 Å, corresponding to the (211) and (312) crystal planes of CuCN (Figure 1e). To further demonstrate the composition of Cu/CuCN, the EDS mapping displays a proportional composition and relatively even distribution of Cu, C, and N, as presented in the inset of Figure 1d and Table S1. After further calcination at 800 °C, the PXRD peaks of CuGT completely disappeared. The PXRD pattern of as-made Cu/CuCN (Figure 1f) suggests that the spindle-shaped substance is a composite material of Cu and CuCN.

**Figure 2:**
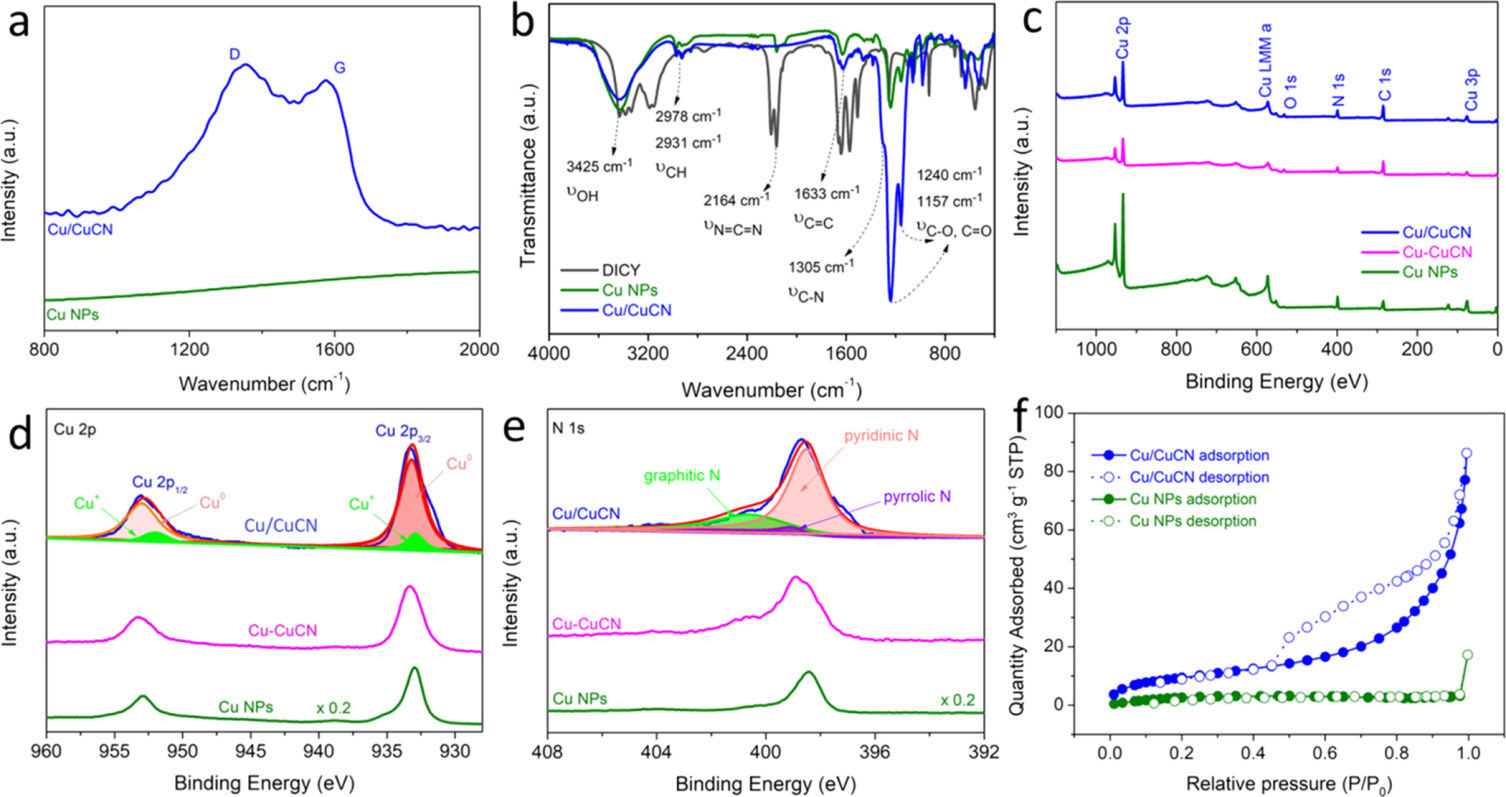
Structural characterization and electronic properties of the prepared fresh Cu/CuCN catalyst. (a) Raman spectra and (b) infrared spectra of the as-prepared Cu NPs and Cu/CuCN samples. (c) XPS survey, comparisons of high-resolution (d) Cu 2p and (e) N 1s XPS spectra between Cu NPs, Cu–CuCN, and Cu/CuCN. (f) N_2_ adsorption–desorption isotherm of the samples.

Raman spectroscopy is a powerful tool for analyzing the micro–nanostructural properties of carbon-based related materials. The Raman spectrum of Cu/CuCN in Figure 2a shows a D band at 1348 cm^–1^ from disordered sp^2^ carbon and a G band at 1575 cm^–1^ from ordered sp^2^ carbon, further evidencing the formation of graphitic carbon materials.^48^ Infrared spectroscopy was carried out to complement the chemical construction of the composite. As shown in Figure 2b, the bending vibration peak of N=C=N at 2164 cm^–1^ disappeared after calcination, as compared to the spectrum of dicyandiamide (DICY). Meanwhile, the vibration signals of C=N and C–N became stronger, which confirmed the formation of CuCN. The thermogravimetric–mass analysis (TG-MS) curves (Figure S17) show that the weight losses of Cu NPs and Cu/CuCN at 800 °C were 90.7 and 84.9%, respectively. Since the excess dicyandiamide provided C≡N^·^ free radicals at high heat conditions, the Cu ions on the surface of the CuGT nanosheets were very reactive, forming a stable linear structural crystal. All these results demonstrated that Cu/CuCN was constructed successfully.

To gain further insight into the chemical elements and evaluate the valance states of the products, X-ray photoelectron spectroscopy (XPS) was performed (Figure 2c–e). The XPS survey displays that the samples are composed of Cu, C, N, and possibly O elements (Figure 2c). The C 1s XPS illustrated the peak at 284.8 eV assigned to sp^3^-hybridized carbon atoms in C–C bonds (Figure S18). The high-resolution XPS signals of Cu 2p_3/2_ and Cu 2p_1/2_ located at 933.2 and 952.9 eV can be attributed to Cu^0^ species, as shown in Figure 2d.^49^ In addition, the binding energy of Cu 2p_3/2_ (932.8 eV) and Cu 2p_1/2_ (952.1 eV) suggested the presence of Cu^+^ on the surface.^50^ Meanwhile, there are no obvious “shake-up” peaks in the higher binding energy region, indicating that Cu^2+^ does not seem to be present in the as-prepared sample.^51^ From analysis results, it is inferred that the copper valence state of Cu/CuCN was mainly 0 and +1. The higher binding energy (∼0.4 eV) of Cu 2p in Cu/CuCN demonstrated the electron accumulation on interfaces. The N 1s spectrum in Figure 2e reveals the existence of pyridinic N (398.5 eV), pyrrolic N (399.2 eV), and graphitic N (400.7 eV).^52^ Among them, pyridine N usually acts as sites to anchor Cu atoms to obtain a Cu–N structure. Moreover, the high-resolution O 1s spectrum further confirmed that the binding energy at 532.1 eV probably belonged to the chemisorbed hydroxyl oxygen and surface C–O,^53^ according to previous studies (Figure S19). The BET specific surface area of Cu/CuCN was calculated to be 34.8 m^2^ g^–1^ based on the N_2_ adsorption–desorption isotherm plot, which was about three times that of Cu NPs (11.3 m^2^g^–1^), as displayed in Figure 2f. Moreover, we draw a conclusion that carbon may inhibit the aggregation of CuCN nanoparticles and further contribute to the specific surface area of Cu/CuCN. This suggested that the formation of heterostructured Cu/CuCN increased the number of active sites for electrocatalysis.

### Theoretical Simulations

**Figure 3:**
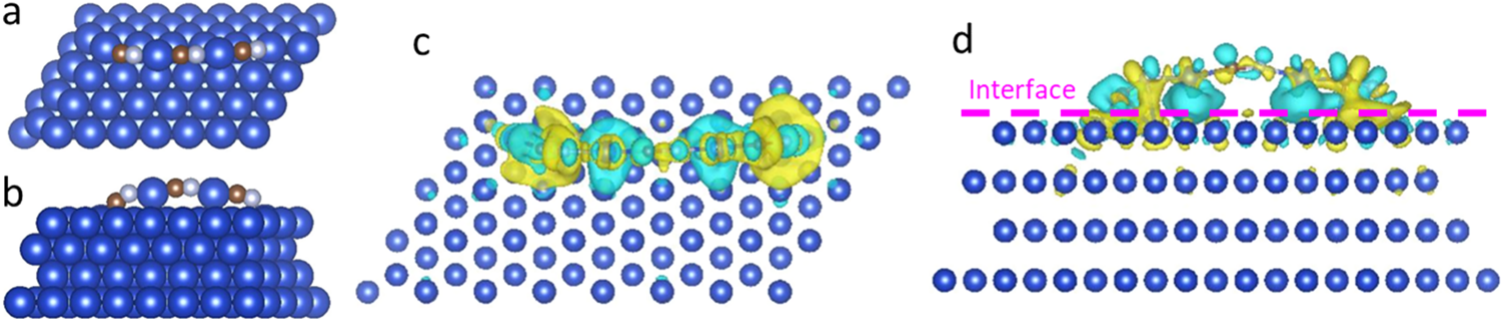
(a, b) Optimized structures of Cu/CuCN. (c, d) Charge density distribution mapping of Cu/CuCN. Yellow represents the electron-rich area and cyan is for the electron-loss area. The isosurface value is 0.005 e Å^–3^.

DFT was applied to explore the distribution of charge density difference at different catalytic surfaces. Images shown in Figure S20 and Figure 3a,b were representative of the most energetically stable configurations of bare copper and Cu-supported CuCN, respectively. We considered that the epitaxial growth of CuCN could largely influence the charge redistribution on the heterointerface of Cu/CuCN. Apart from that, to take into account the electronic effect of CuCN, we calculated the charge density difference of Cu(111)/CuCN and Cu(111) and observed that the obvious charge redistribution emerged at the interface district, as shown in Figure 3c,d. The results clearly illustrated that the heterostructured Cu/CuCN had yielded a charge density enhancement on CuCN, while the opposite was true on copper substrates. Furthermore, Bader charge analysis indicated that there was an obvious electron migration trend transferred from Cu, which is on the surface of the underlying Cu to C or N of the CuCN interface with sufficient active sites.^54^ This accelerated the electron transfer and thus improved the water oxidation activity. After calculating the free energy plots, it is further proven that the oxygenated intermediate difference is about 3.39 eV for Cu/CuCN with the novel heterointerface in acid solution, which was beneficial for OER (Figure S21). This was in line with previously reported results.^55^

### Electrocatalytic Performance Evaluation of Cu/CuCN under Alkaline Conditions

**Figure 4:**
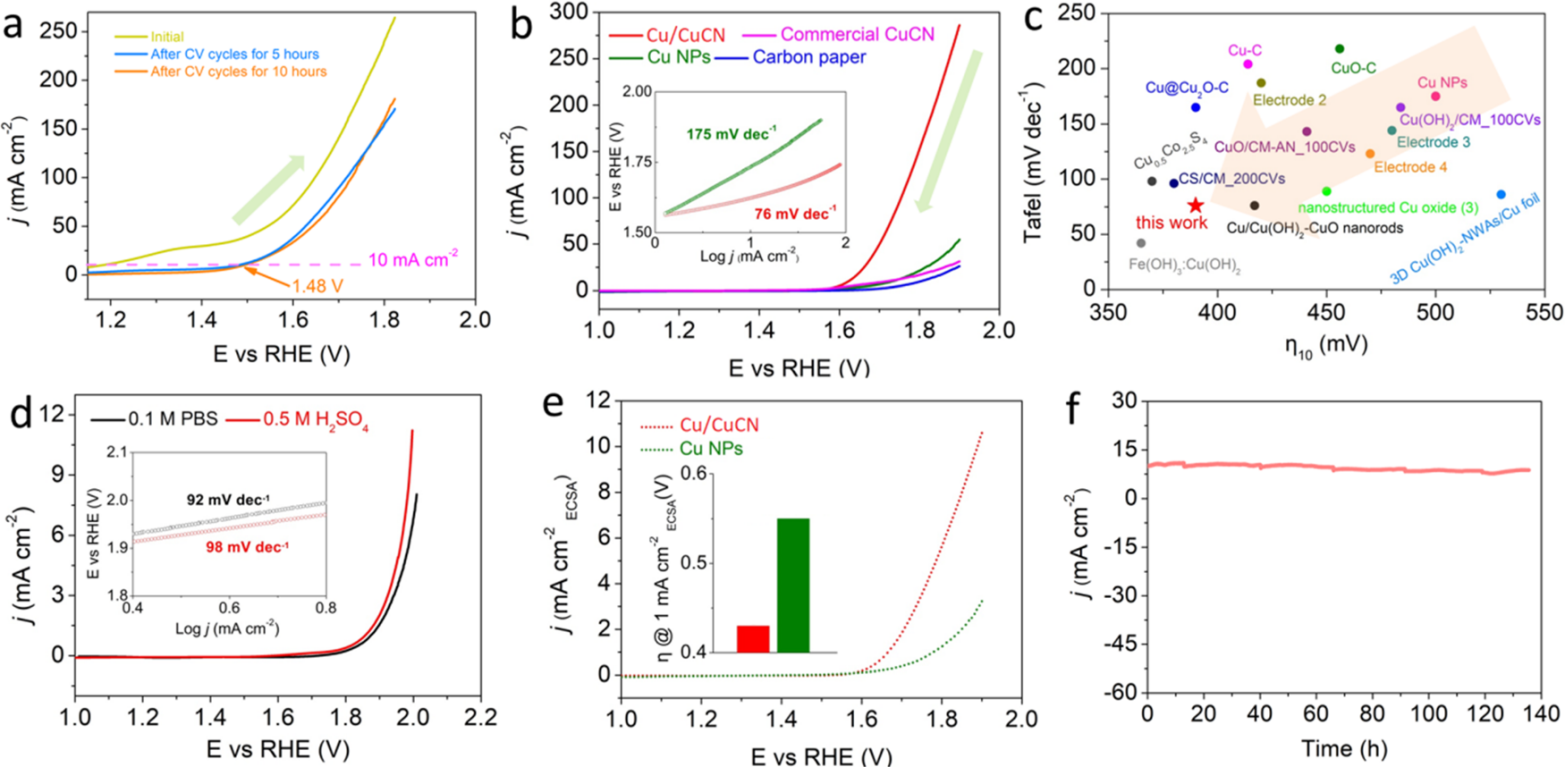
Evaluation of electrocatalytic performance of Cu/CuCN for OER. (a) Forward scanning OER polarization curves of Cu/CuCN in 1.0 M KOH on a carbon paper electrode with a 5 mV s^–1^ scanning rate (green arrow). (b) Backward scan OER polarization (green arrow) and inset of panel (b): corresponding Tafel plots of Cu/CuCN and control samples. (c) Comparison of both the Tafel slope and catalytic activity with references. (d) OER polarization curves and derived Tafel plots of Cu/CuCN measured in 0.5 M H_2_SO_4_ and 0.1 M PBS. (e) Normalized OER polarization curves of Cu/CuCN and Cu NPs by ECSAs. (f) *i*–*t* curve of Cu/CuCN catalysts under an initial current density of 10 mA cm^–2^, demonstrating the unprecedented high stability of Cu/CuCN.

The OER performances of different samples were investigated in 1.0 M KOH solution with N_2_-saturated solutions with a common three-electrode device. As expected, Cu/CuCN showed extraordinary OER activity, with 10 mA cm^–2^ (η_10_) at 250 mV after CV cycles for 10 h (without *iR* correction) in the forward scan (Figure 4a). Furthermore, the almost unchanged CV cycle curves from 5 to 10 h demonstrate that the obtained Cu/CuCN is in a stable state for use in OER testing. However, under certain circumstances, when the oxidation peak (such as Ni, Fe, Co, etc.) appears in the polarization curves, it will affect the true current signal of the catalyst to assess the “onset potential” and η_10_. We suggest that a backward scan is employed to reasonably evaluate the catalyst performance, as previously reported in the literature (Figure S22).^56^ Therefore, we mainly use the backward scanning curve to evaluate the water oxidation activity in this article. As shown in Figure 4b, the Cu/CuCN sample offers 10 mA cm^–2^ of current density at only a 380 mV overpotential (without *iR* correction), while the Cu nanoparticles (NPs) need a much larger overpotential of 600 mV. In addition, the obtained catalyst can drive higher current densities (200 mA cm^–2^) under much lower overpotentials (∼600 mV), compared to those of reported Cu-based catalysts. As expected, the Cu/CuCN samples exhibited the best OER performance compared to Cu NPs and carbon paper, and the OER activity of Cu/CuCN could be optimized by adjusting the ratio of CuGT to dicyandiamide (Figure S23). In addition, we also explored the effect of different calcination temperatures and the precursor of the Cu(NO_3_)_2_-based catalyst for OER (Figures S24–S27). The results revealed that the Cu/CuCN samples had superior electrocatalytic activity. These results confirmed that the heterostructured Cu/CuCN catalyst was an active catalyst for water oxidation. To clearly describe the OER kinetics, the Tafel slope was obtained. Notably, the kinetics of OER inversely depend on the value of the Tafel slope, and the lower the slope value, the faster the OER kinetics.^57^ As shown in the inset of Figure 4b, Cu/CuCN exhibited a much smaller slope of 76 mV dec^–1^ than Cu NPs (175 mV dec^–1^), which even matched that of noble metal Ir/C and outperformed the previously reported Cu-based catalysts in alkaline media, suggesting that OER at Cu/CuCN followed a favorable reaction trend with faster kinetics. Details on comparisons including η_10_ and the Tafel slope are summarized in Figure S28. As shown in Figure 4c, our Cu/CuCN catalyst exhibited superior OER performance to other available Cu-based OER catalysts in alkaline environments (Table S2). In detail, Cu/CuCN showed much smaller Tafel slope values than the reported Cu-based catalysts in potassium hydroxide solution. In addition, the needed overpotential (η_10_) is also substantially lower than those of most non-precious Cu-based OER catalysts and other OER catalysts, including the recently reported excellent electrocatalysts Cu(OH)_2_/CM_100CVs (484 mV), nanostructured Cu oxide (400 mV), 3D Cu(OH)_2_-NWAs/Cu foil (530 mV), Cu–C (414 mV), annealed CuO-3 (580 mV), exfoliated NiFe LDHs (300 mV), MoS_2_-Ni_3_S_2_ HNRs/NF (249 mV), IrO_2_/NF (285 mV), and so forth (see the details in Table S2). The exceptional electrocatalytic behavior toward water oxidation is primarily due to the synergy of rich CuCN active species and better conductivity of the Cu support. We also explored the OER processes of the Cu/CuCN electrode in different concentrations of potassium hydroxide solution (Figure S29). In addition, the superb performances of Cu/CuCN toward OER were also studied in 0.5 M H_2_SO_4_ and 0.1 M PBS, respectively. It should be pointed out that Cu/CuCN also demonstrated excellent activity accompanied by a low Tafel slope of 98 (0.5 M H_2_SO_4_) or 92 mV dec^–1^ (0.1 M PBS), as shown in Figure 4d. We compared the OER results of Cu/CuCN including the Tafel slope and η_10_ under 0.5 M H_2_SO_4_ and 0.1 M PBS conditions (Figure S30). Impressively, Cu/CuCN exhibited superior OER activities over a wide pH range and further was also the first case of a Cu-based water oxidation catalyst in pH-universal electrolytes.

Additionally, double-layer capacitance (*C*_dl_) measurements were employed to evaluate the electrochemically active surface area (ECSA), and results revealed the larger *C*_dl_ of Cu/CuCN (1.6 mF cm^–2^) compared to that of Cu NPs (1.0 mF cm^–2^) (Figure 4e and Figure S31), suggesting a larger exposed surface area constructed on Cu/CuCN. For confirmation, the ECSA-normalized polarization curves might help to clearly uncover the intrinsic activity of catalysts. Obviously, the ECSA-normalized value of Cu/CuCN was larger than that of Cu NPs (Figure 4e), indicating the higher OER performance by the synergistic effect of CuCN and Cu supports. Moreover, Cu/CuCN showed a much higher current density in acidic solution than in neutral conditions, implying faster OER kinetics in 0.5 M H_2_SO_4_ media (Figure S32). The fast OER reaction kinetics of Cu/CuCN was reflected by a lower resistance of 7.0 Ω at 1.53 V vs RHE (Figure S33), while the charge transfer resistance was 9.0 Ω for Cu NPs.

Apart from the excellent OER performance, stability is also an essential factor to be considered for a superior catalyst. Furthermore, the chronoamperometric curves of the Cu/CuCN electrode for water oxidation were examined at a constant current density of 10 mA cm^–2^ for 136 h, as shown in Figure 4f. The durability test was performed as shown in Figure S34, which shows that the activity of Cu/CuCN only exhibited slight degradation after a 40 h run, suggesting its high durability. Impressively, the overpotentials just added 40 mV with the time extended to 136 h. The results meant that Cu/CuCN catalysts had superior long-time stability in alkaline water oxidation. In addition, the long-term durability revealed the better durability in acid and neutral environments (Figure S35). The results of PXRD and XPS after 1000 cycles also demonstrated that the formation of Cu_2+1_O species originated from the partial oxidation of CuCN during the OER test (Figure S36).^58,59^

### Characterizations of Samples after 136 h of OER and Proposed OER Mechanism

**Figure 5:**
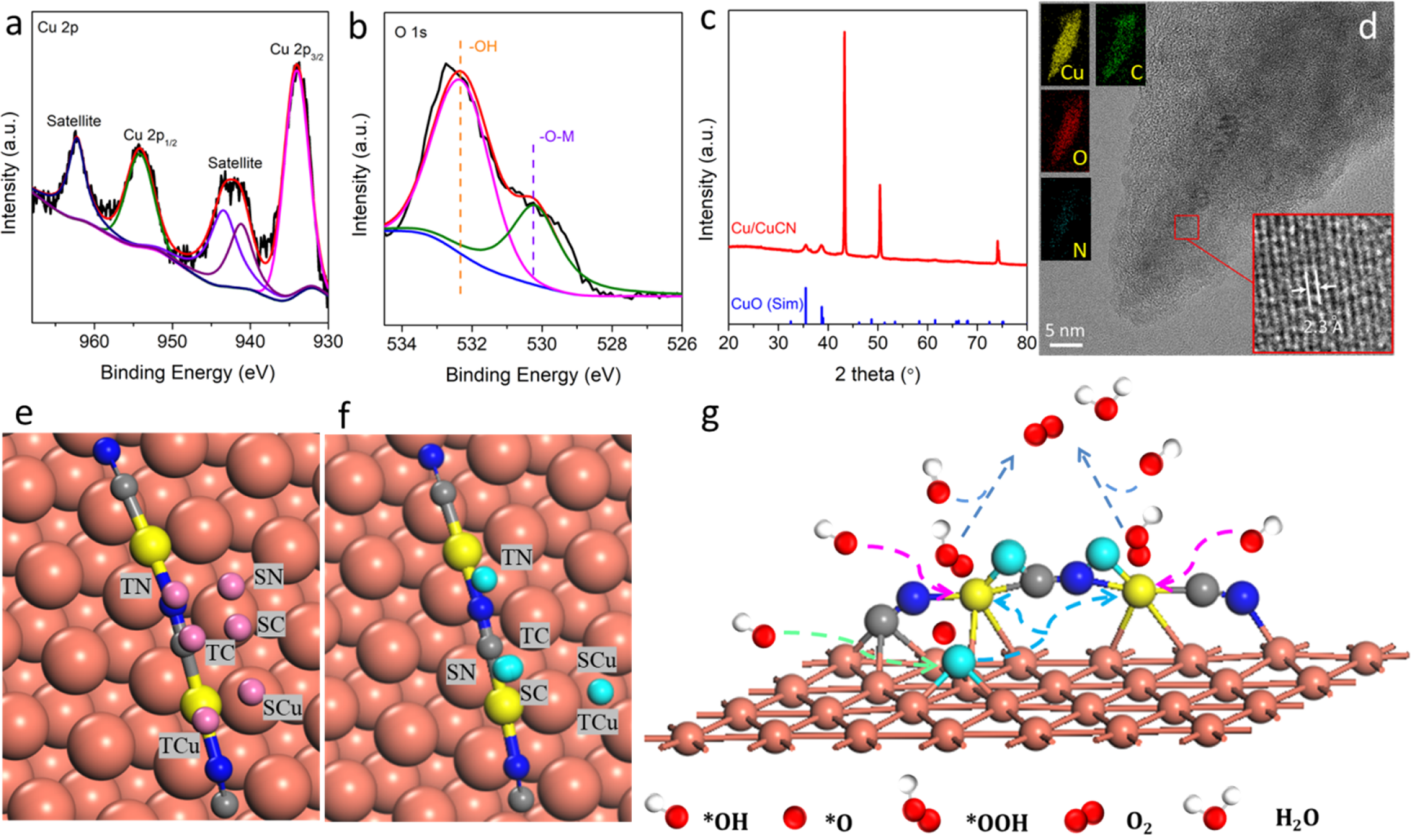
Post-characterizations of the Cu/CuCN samples after 136 h of OER in an alkaline environment. High-resolution XPS spectra of (a) Cu 2p and (b) O 1s. (c) PXRD pattern. (d) HRTEM images of EDX elemental mapping of Cu, C, O, and N of Cu/CuCN. Optimized configurations of (e) Cu/CuCN and (f) Cu/CuCN with CuO species chemisorption of *O intermediates. Blue = N, gray = C, brown and yellow = Cu, pink = adsorbed O (before reaction), and cyan = adsorbed O (after reaction). Cu, C, and N represent copper, carbon, and nitrogen elements, respectively. T and S stand for top and side sites, respectively. (g) Illustration of the potential water oxidation reaction mechanism.

To further reveal the reaction mechanism for water oxidation under alkaline conditions, Cu/CuCN was characterized by XPS, PXRD, Raman, FESEM, TEM, and HRTEM investigations after 136 h of OER cycles, as depicted in Figure 5. Cu XPS spectra showed the existence of two 2p and satellite peaks corresponding to the Cu–O bonds (Figure 5a).^60^ The O 1s spectrum (Figure 5b) further verified the generation of Cu–O species after 136 h of OER. Moreover, the spectra of C and N 1s for catalysts after 136 h of OER displayed no noticeable change, compared to those of the fresh catalysts (Figure S37). The PXRD pattern for post-OER (Figure 5c) showed that the diffraction peaks at 35.5 and 38.7° matched the (002) and (111) planes of copper oxide, respectively, proving the existence of copper oxide.^61^ However, the diffraction peaks of CuCN have obviously disappeared. The possible reason was that during OER, some of the oxygens are intercalated into the CuCN lattice, causing it to become more disordered. In addition, the FESEM image (Figure S38a) of Cu/CuCN showed that it retained the pristine heterostructures with a relatively rough surface without deviation, demonstrating the overall stability of the material. In contrast to that of the fresh catalysts, the Raman spectrum had hardly changed, as shown in Figure S38b. According to the analysis of TEM and HRTEM images in Figure 5d, the fringe spacing of 0.23 nm matched the (111) lattice plane of CuO, suggesting the formation of the CuO phase on the surface of the Cu/CuCN catalyst after a long-term OER test. Meanwhile, the corresponding elemental mapping results (inset of Figure 5d) verified the Cu, C, N, and O uniform distribution across the Cu/CuCN heterostructures. All these observations indicated the rapid surface reconstruction in the presence of copper oxide and CuCN, *in situ* transforming from crystalline into amorphous oxygen intercalated CuCN. Frankly speaking, the new active species of the interface could boost the water oxidation conversion kinetics during the electrocatalytic process.

To verify whether O finds it facile to intercalate into CuCN and the vital role of CuO in alkaline OER, we provided an insight of how the heterostructures of CuCN embedded on Cu respond to the O atom at the atomic level through DFT calculations. The DFT calculations optimized the O atom to be near Cu/CuCN at various initial sites (top and side sites for Cu, C, and N) on the right top of CuCN and the side of CuCN. Figure 5e,f demonstrates that in most cases, especially when the initial positions are close to regardless of C or N being on the side or top of Cu/CuCN, the O atom spontaneously intercalates between Cu and C(N). However, when the initial position of O atom closed to Cu, it energetically tends to be stabilized at sites where pure Cu is located, far away from CuCN. The result clearly suggested that the formation of oxygen-embedded CuCN is facile, once the O atom is near to CuCN, which is consistent with the above experimental characterization after 136 h of OER. Previous studies have shown that an interface between the active species and substrate can also significantly promote the water oxidation activity by decreasing the Gibbs free energy.^21^ Therefore, the formation of an amorphous oxygen-embedded Cu/CuCN and Cu-based oxide layer may also affect the interface Gibbs free energy and guarantee the catalytic activity enhancement.^62^ Moreover, as illustrated in Figure 5g, the proposed OER mechanism demonstrated that the adsorption reactions were no more limited to single sites, while it was the synergistic effect under the action of multiple active sites. First, the *OH species were adsorbed on CuO sites (green arrows) and were transformed into *O species. Then, the obtained *O species could selectively migrate to sites of Cu–(O)–CN (baby blue arrows). Hereafter, the rapid generation of *OOH species broke the energy barrier and accelerated the OER reaction (pink arrows). Lastly, oxygen molecules were obtained through the process of *OOH deprotonation (dark blue arrows). From another perspective, the orbital hybridization of Cu 3d and O 2p illustrated in Figure S39 also gave a reasonable explanation of the superior OER activity, which is due to the electronic regulation of CuCN from the oxygen intercalated and CuO species. Generally, the antibonding orbital state for optimized adsorption influences the catalytic performance of the catalyst, whereas the bonding states are fully filled and are usually far below the Fermi level (*E*_F_).^63,47^ Combined with the distribution of charge density differences and the above analysis, when more electrons fill the antibonding orbitals, we supposed that the activation of water molecules and intermediates (*OH and *OOH) becomes easier at the interface. Therefore, the heterointerface-induced charge transfer substantially triggers the regulation of OER activity.

Based on the above analysis, the good performance of Cu/CuCN catalysts can be attributed to the following aspects: (1) the better intrinsic metallic property of the copper substrate ensures the high electron conductivity and benefits the formation of mainly active sites of CuO species; (2) the reinforced electronic interaction and the effective charge transfer of the copper substrate and copper oxide; and (3) the unique interfaces were useful for electrocatalytic reactions. More importantly, we highlighted the essential role of the design and engineering of a heterojunction with rich active sites by the aforementioned series of experimental and theoretical analyses.

### Electrocatalytic Overall Water Splitting of Cu/CuCN under Alkaline Media

**Figure 6:**
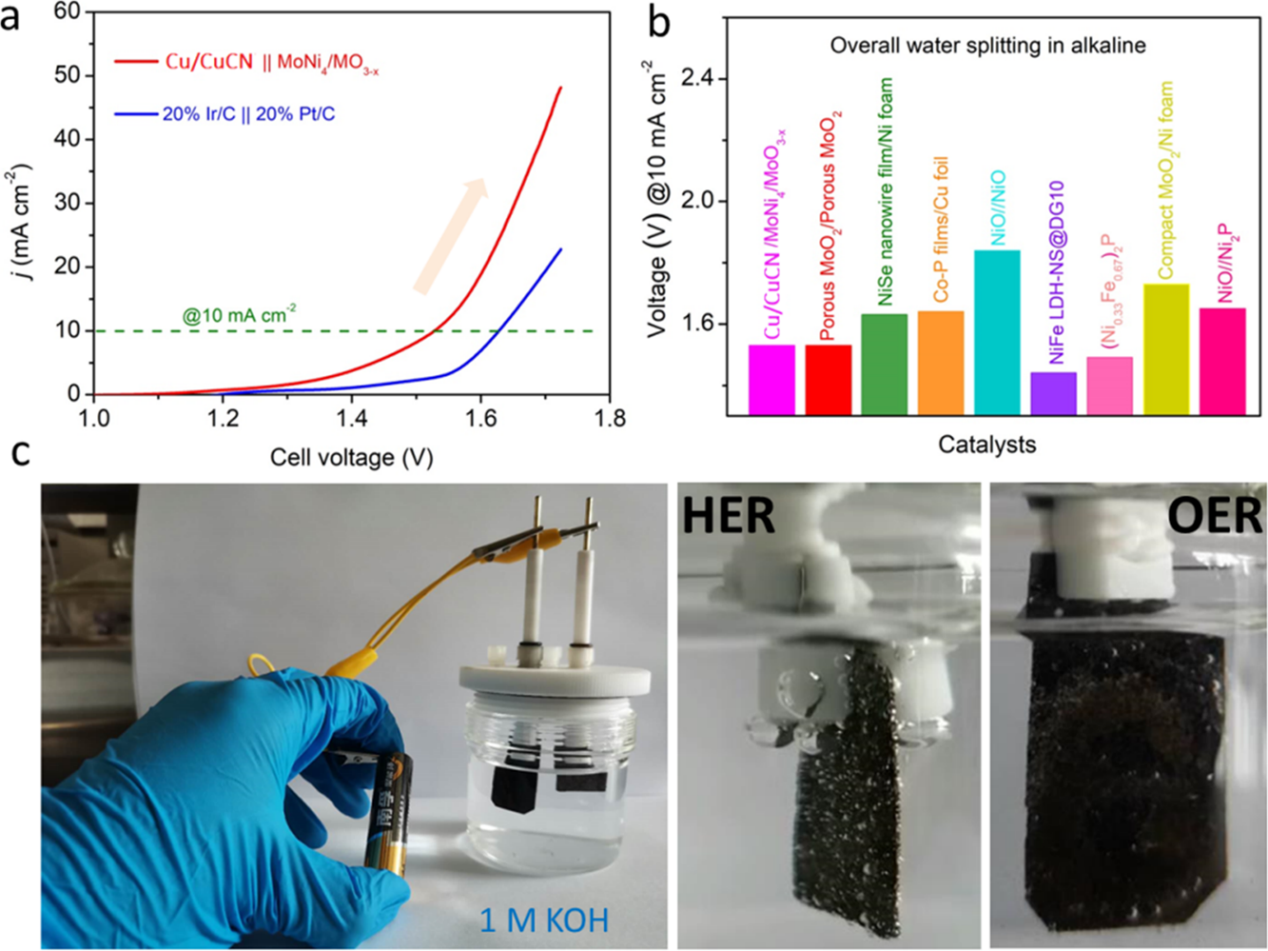
**(**a) Polarization curves of Cu/CuCN||MoNi_4_/MoO_3–*x*_ and 20% Ir/C||Pt/C catalyst couples for the splitting of water. (b) Comparison of Cu/CuCN||MoNi_4_/MoO_3–*x*_ with other reported catalysts for electrocatalytic activity. (c) Optical photos of water splitting driven using a 1.5 V battery.

Based on the excellent alkaline OER results aforementioned, we anticipated that Cu/CuCN could act as an anode electrocatalyst coupling with the reported superior MoNi_4_/MoO_3–*x*_ cathode for overall water splitting.^43^ Hence, a two-electrode configuration was employed (Figure 6a). The results exhibited excellent alkaline water splitting demanding only 1.53 V at 10 mA cm^–2^. Impressively, the water splitting activity of this electrolyzer is better than that of the noble metal 20% Ir/C||Pt/C catalysts. It is remarkable that our catalyst is comparable to reported non-noble metal catalysts for water splitting in alkaline media (Figure 6b and Table S3). In addition, a two-electrode Cu/CuCN and MoNi_4_/MoO_3–*x*_ device was successfully driven by a 1.5 V battery, as shown in Figure 6c. Interestingly, the evolution of both oxygen and hydrogen bubbles could be clearly observed. In addition, the long-term water splitting stability indicated that Cu/CuCN||MoNi_4_/MoO_3–*x*_ had superior durability in 1.0 M KOH solution (Figure S40). Our findings clearly demonstrate that the superior activity for water splitting of our heterostructured low-price metal electrocatalysts signifies their great potential to exchange noble metal catalysts for sustainable H_2_ production.

## Conclusions

In summary, we had demonstrated an efficient epitaxial growth approach for the creation of the Cu/CuCN heterostructure. When applied in the OER in pH-universal conditions, the Cu/CuCN pre-catalyst exhibited very high catalytic activity, especially easy driving at10 mA cm^–2^, showing a relatively small overpotential (250 or 380 mV) and Tafel slope (76 mV dec^–1^) with an extremely long operational stability (136 h OER run) in an alkaline medium. Experiments combined with theoretical simulations confirmed *in situ*-formed oxygen-embedded CuCN and CuO active species during alkaline OER. We concluded that the interfacial active sites played a vital role in the optimized adsorption and conversion of the OER intermediates. As a result, Cu/CuCN coupling with the MoNi_4_/MoO_3–*x*_ catalyst can be started up using a battery of 1.5 V in 1.0 M KOH, which is also an overall water splitting record for Cu-based catalysts. This work demonstrated a promising heterostructure modulation approach to fabricate other heterojunctions for broader applications.

## Supporting Information Available

Synthesis, characterization, theoretical calculations, and electrochemical tests of Cu/CuCN materials; PXRD, TG-DSC curves; SEM and TEM images; XPS spectra; CV curves; ECSA and *C*_dl_; long-term stability test, and summary of recently reported electrocatalysts for the OER and overall water splitting (PDF)
