# Supplementary material for: Stable, Efficient, Copper Coordination Polymer-Derived Heterostructured Catalyst for Oxygen Evolution under pH-Universal Conditions
Source: ACS Appl Mater Interfaces. 2021 May 21;13(21):25461–71. doi: 10.1021/acsami.1c01424 (PMC8289192; doi:10.1021/acsami.1c01424)
Supplement: Supplementary file 1 — am1c01424_si_001.pdf [file am1c01424_si_001.pdf]

Supporting Information

Stable, Efficient, Copper Coordination Polymer-derived Heterostructured Catalyst for Oxygen Evolution under pH-universal Conditions

Ligang Wang[1], ‡ Ning Ma[1,2], ‡* Nian Wu[3], ‡ Xiaoge Wang[1], Junjie Xin[1], Dingsheng Wang[4], Jianhua Lin[1], Xingguo Li[1], and Junliang Sun[1]*

1 College of Chemistry and Molecular Engineering, and Beijing National Laboratory for Molecular Sciences (BNLMS) 5 Yiheyuan Road, Peking University, Beijing 100871, P. R. China.

2 Hubei Key Laboratory of Polymer Materials, Key Laboratory for the Green Preparation and Application of Functional Materials (Ministry of Education), Hubei Collaborative Innovation Center for Advanced Organic Chemical Materials, School of Materials Science and Engineering, Hubei University, Wuhan 430062, P. R. China.

3 Institute for Interdisciplinary Information Sciences, Tsinghua University, Beijing, 100084, P. R. China.

4 Department of Chemistry, Tsinghua University, Beijing, 100084 China.

Corresponding Author

E-mail address: *junliang.sun@pku.edu.cn (J.L.Sun); *ningma@pku.edu.cn (N.Ma).

Table of Contents

Figure S1. Powder X-ray diffraction patterns of obtained Cu/CuCN precursor.
Figure S2. The SEM images of samples from the various ratios of CuGT to D.
Figure S3. SEM results of Cu NPs from CuGT after 800 ℃ calcination.
Figure S4. TEM image of Cu NPs from CuGT after 800 ℃ calcination.
Figure S5. Powder X-ray diffraction patterns of Cu NPs from CuGT at 800 ℃ calcination.
Figure S6. SEM results of Cu NPs-1 and Cu/CuCN-1 at 700 ℃ calcination.
Figure S7. Powder X-ray diffraction results of prepared Cu NPs-1 and Cu/CuCN-1.
Figure S8. SEM results of prepared Cu NPs-2 and Cu/CuCN-2 at 600 ℃ calcination.
Figure S9. Powder X-ray diffraction results of prepared Cu NPs-2 and Cu/CuCN-2.
Figure S10. SEM results of prepared Cu/CuCN catalyst.
Figure S11. SEM of obtained $Cu(NO_3)_2$+Glu+D and its counterparts after 800 ℃ calcination.
Figure S12. PXRD and photograph of $Cu(NO_3)_2$+Glu+D and its counterparts after 800 ℃ calcination.
Figure S13. SEM results of $Cu(NO_3)_2$+Glu+D and its counterparts after 700 ℃ calcination.
Figure S14. PXRD of $Cu(NO_3)_2$+Glu+D and its counterparts after 700 ℃ calcination.
Figure S15. SEM results of $Cu(NO_3)_2$+Glu+D and its counterparts after 600 ℃ calcination.
Figure S16. PXRD patterns of $Cu(NO_3)_2$+Glu+D and its counterparts after 600 ℃ calcination.
Figure S17. Thermal Gravimetric-mass spectra of Cu NPs and Cu/CuCN.
Figure S18. The high resolution XPS spectra of C 1s from obtained three samples.
Figure S19. The XPS spectra of O1s from the Cu/CuCN, Cu-CuCN and Cu NPs samples.
Figure S20. The schematic structures of on a Cu(111) facet.
Figure S21. The calculated OER free energy diagram of the Cu/CuCN catalysts.
Figure S22. The CVs was employed to determine “onset potential”.
Figure S23. OER polarization curves of the obtained catalysts from various ratios of CuGT to D (dicyandiamide).
Figure S24. The OER polarization curves of Cu/CuCN-1 and Cu/CuCN-2 catalysts.
Figure S25. The CVs of Cu/CuCN after different cycles.
Figure S26. The OER polarization curves of $Cu(NO_3)_2$+Glu+D after 800 ℃ calcination in 1.0 M KOH.
Figure S27. The OER polarization curves of catalysts from $Cu(NO_3)_2$+Glu+D after 600 and 700 ℃ calcination.
Figure S28. Detail comparison including Tafel slope and overpotential at 10 mA cm^{-2}.
Figure S29. Water oxidation curves of the Cu/CuCN electrode in 0.1, 1.0 and 3.0 M KOH solutions.
Figure S30. The Tafel slope and overpotential at 1 mA cm^{-2} under 0.5 M H_2SO_4 and 0.1 M PBS solutions.
Figure S31. CV curves and C_{dl} of the prepared catalysts at non-Faraday area.
Figure S32. The obtained current density versus scan rate to assess the C_{dl} of the Cu/CuCN catalysts in 0.5 M H_2SO_4 and 0.1 M PBS solution.
Figure S33. AC impedance of Cu/CuCN and Cu NPs catalysts.
Figure S34. The polarization curves of the Cu/CuCN electrode before and after the stability test in 1.0 M KOH.
Figure S35. Electrochemical stability of the Cu/CuCN electrode under acidic and neutral conditions.
Figure S36. Postcharacterizations of the Cu/CuCN after 1000 cycles acidic and neutral OER.
Figure S37. The XPS spectra of a C1s, and b N 1s from the Cu/CuCN after 136 h OER.
Figure S38. The SEM and Raman spectra from the Cu/CuCN after 136 h OER.

Figure S39. Schematic illustration of orbital hybridization of Cu 3d and O 2p. The colored parts represent the electron filling.
Figure S40. Long-term stability test of Cu/CuCN||$MoNi_4/MoO_{3-x}$ in a two-electrode water electrolysis cell.
Table S1. The atomic ratio of different elements of Cu/CuCN.
Table S2. OER performances of Cu- and other transition metals–based OER electrocatalysts.
Table S3. Comparison of the overall water splitting activities among different non-precious metal electrocatalysts tested under alkaline conditions.

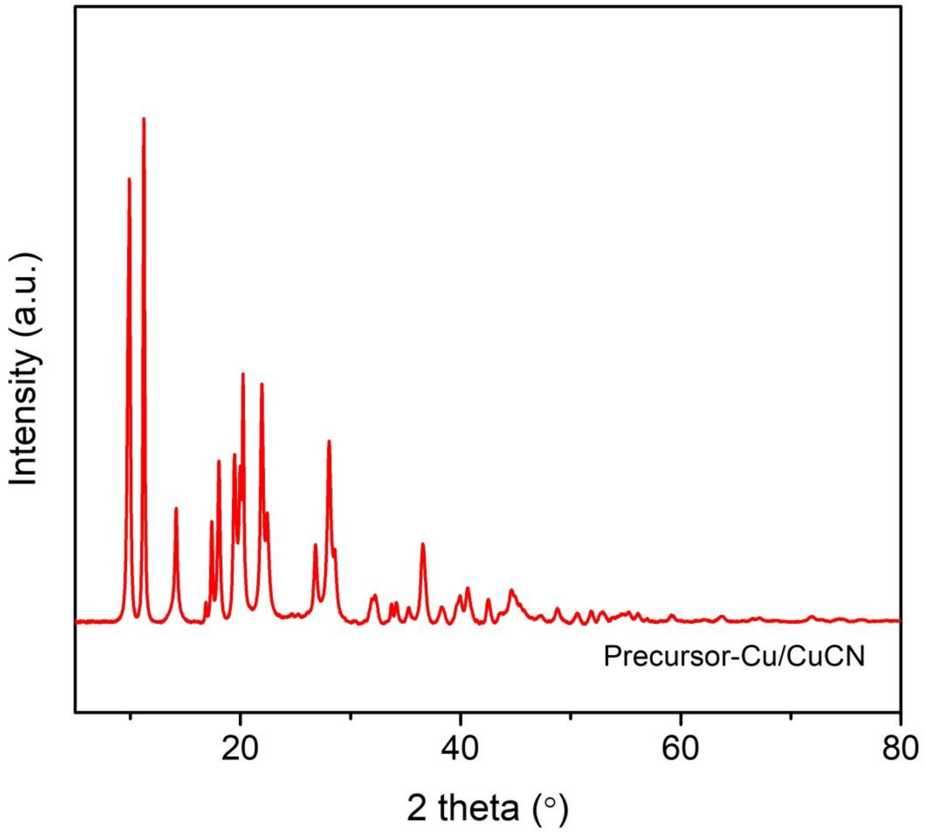

Figure S1. Powder X-ray diffraction patterns of obtained Cu/CuCN precursor.

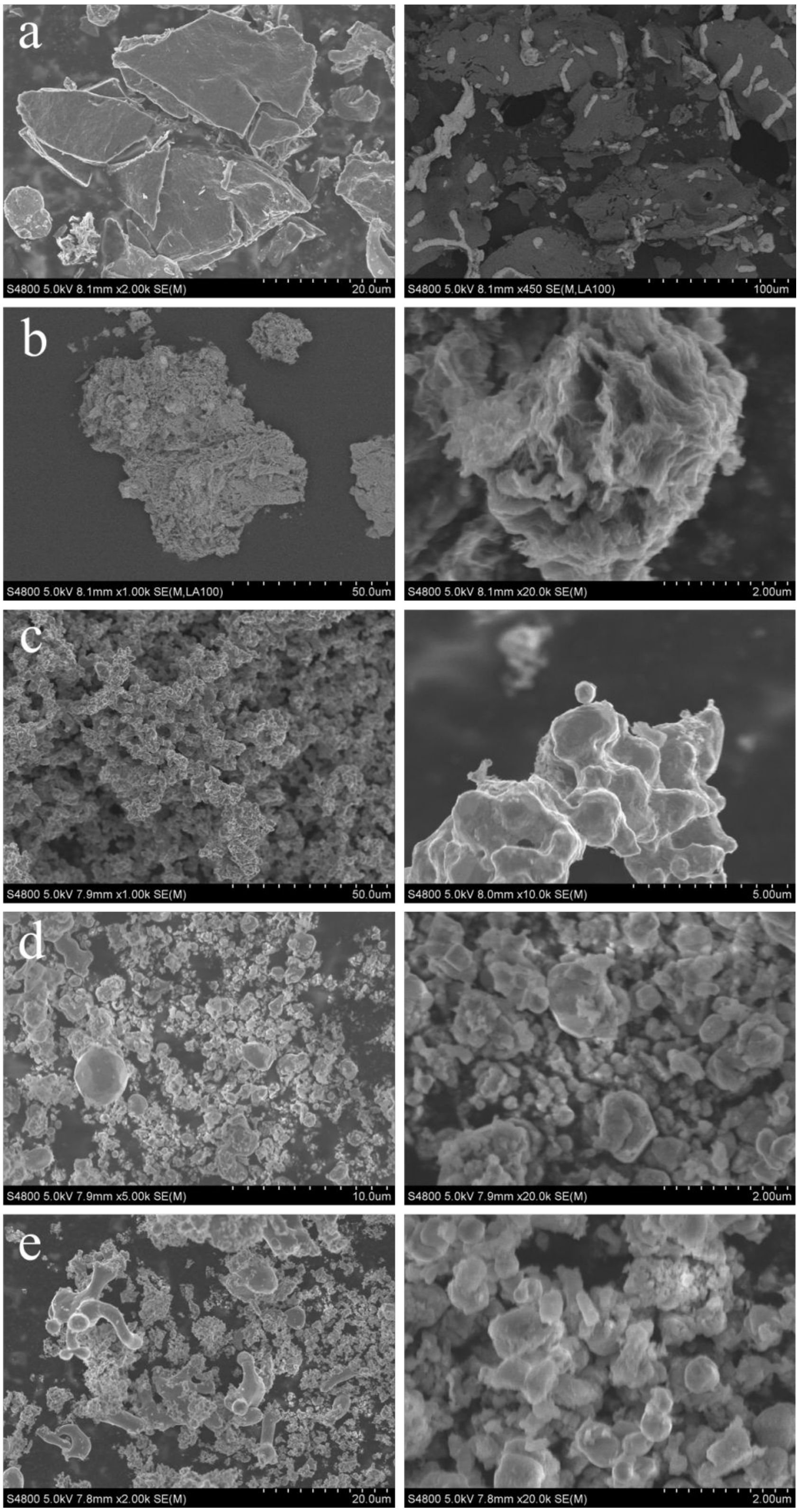

Figure S2. The SEM images of samples from the various ratios of CuGT to D (dicyandiamide): a 1:1, b 1:3, c 1:10, d 1:50, and e 1:100.

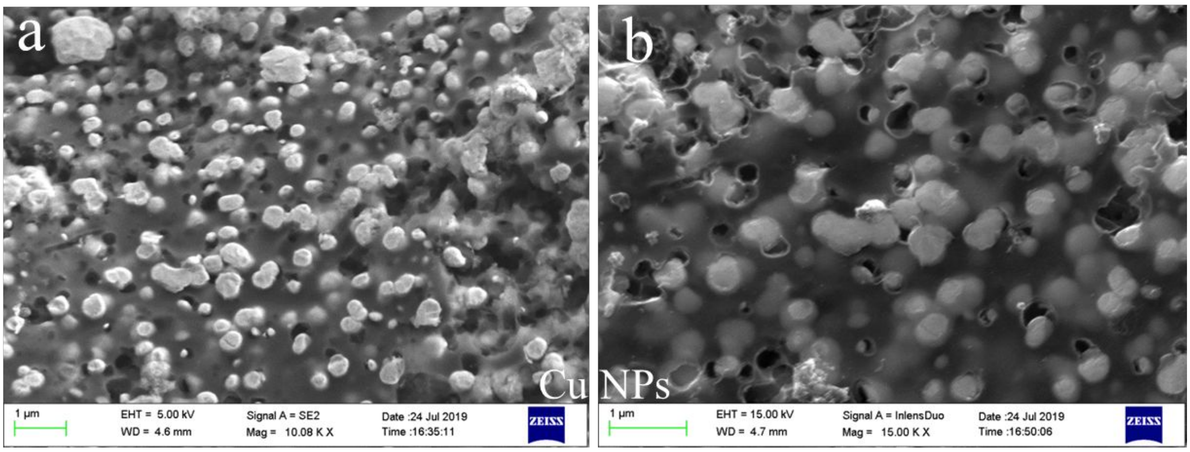

Figure S3. SEM results of Cu NPs (a, b) from CuGT after 800 ℃ calcination. The CuGT as the precursor was calcined at 800 ℃ for 3 h in an argon atmosphere with a ramping rate of 2 ℃ min^{-1}.

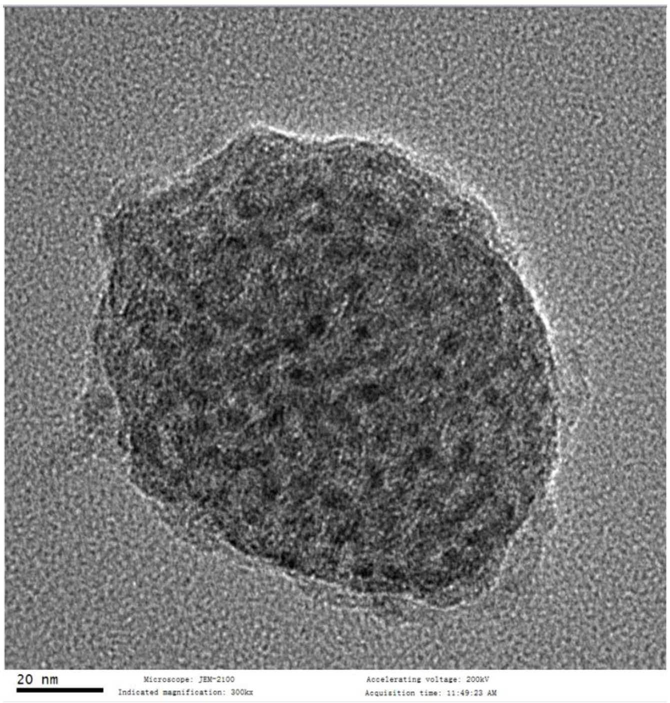

Figure S4. TEM image of Cu NPs from CuGT after 800 ℃ calcination. The obvious Cu nanoparticles were detected.

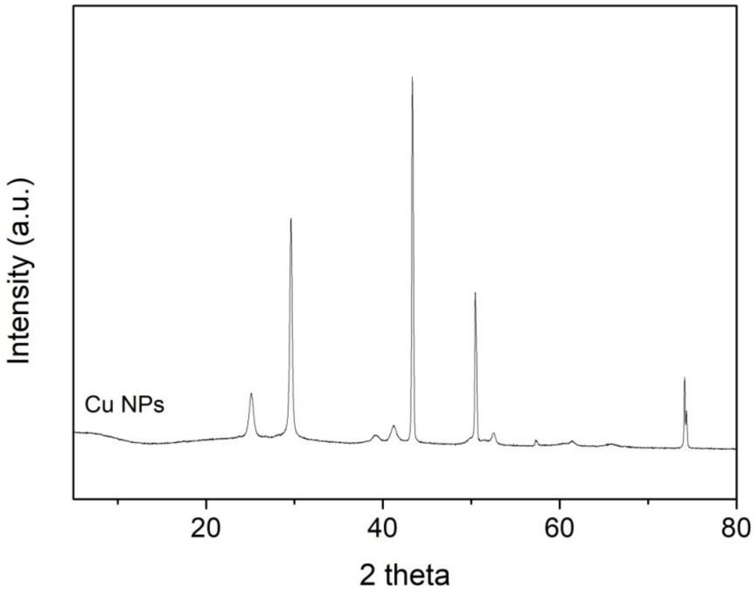

Figure S5. Powder X-ray diffraction patterns of Cu NPs from CuGT at 800 ℃ calcination.

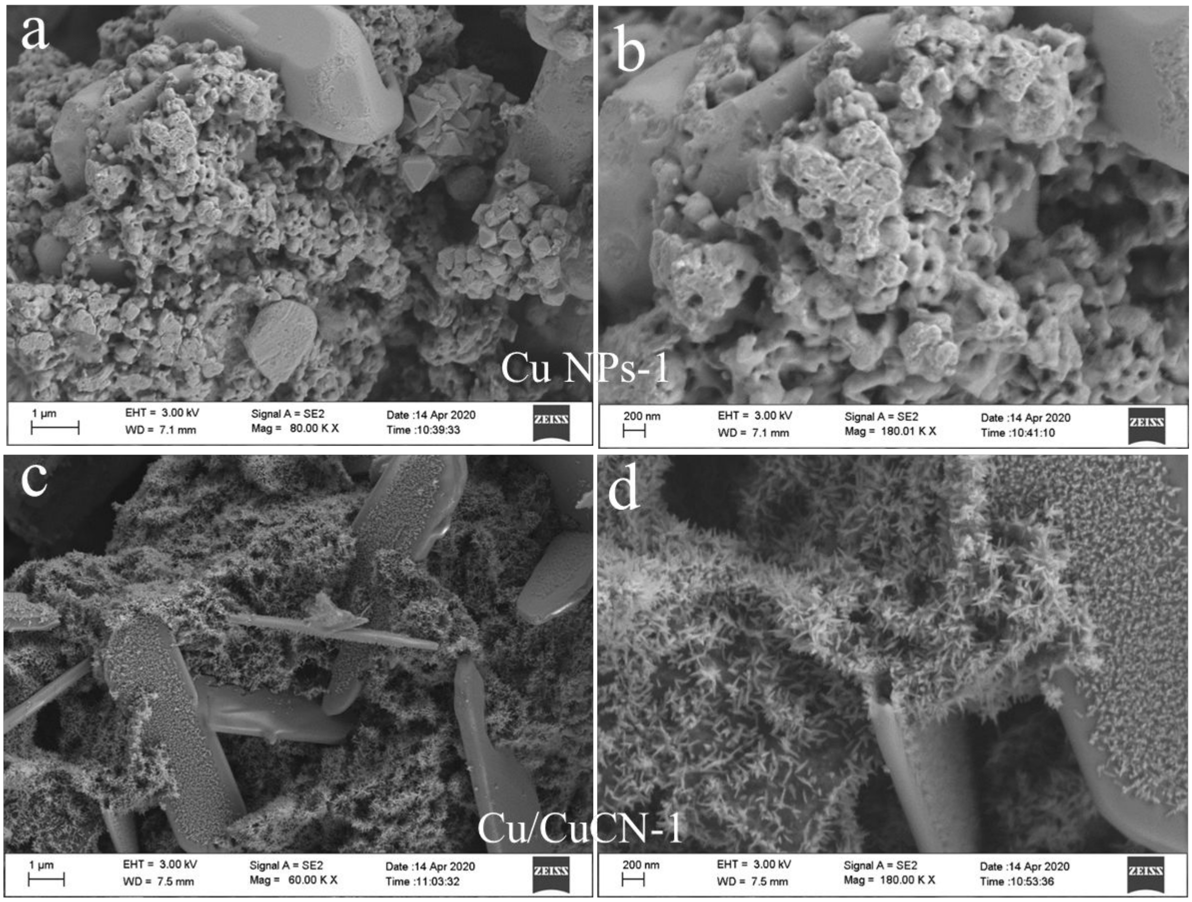

Figure S6. SEM results of Cu NPs-1 and Cu/CuCN-1 at 700 ℃ calcination. (a, b) are Cu NPs-1 and (c, d) are Cu/CuCN-1.

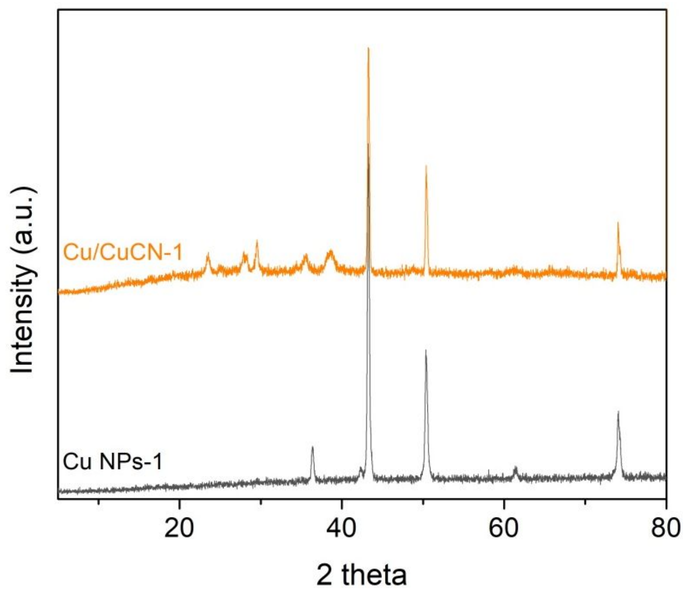

Figure S7. Powder X-ray diffraction results of prepared Cu NPs-1 and Cu/CuCN-1.

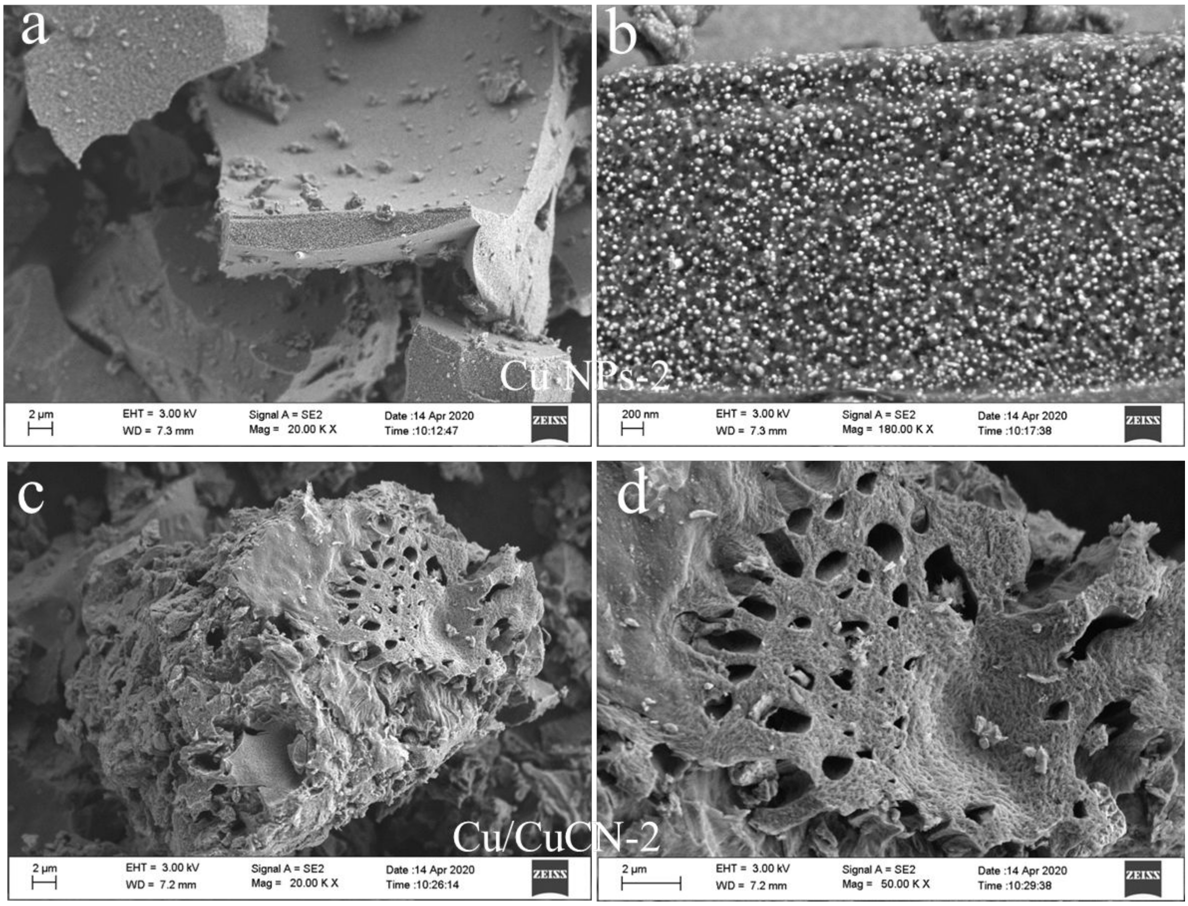

Figure S8. SEM results of prepared Cu NPs-2 and Cu/CuCN-2 at 600 ℃ calcination. (a, b) are Cu NPs-2 and (c, d) are Cu/CuCN-2, respectively.

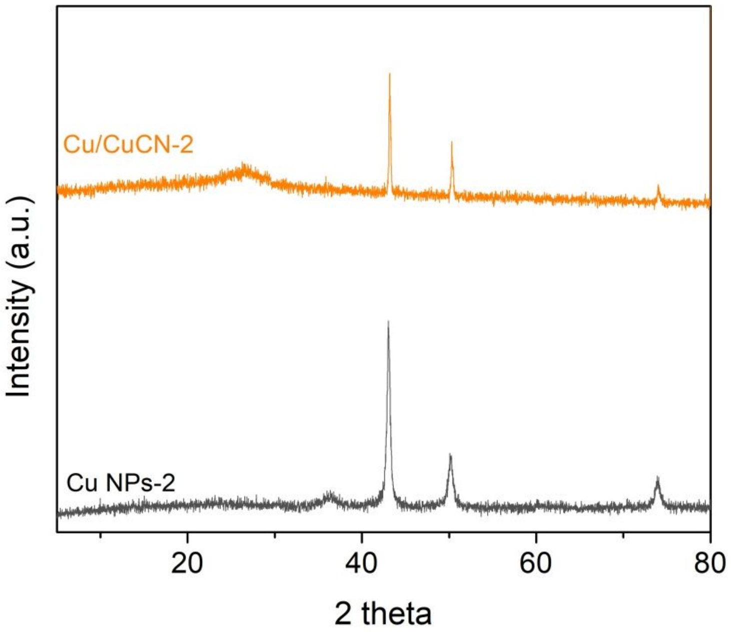

Figure S9. Powder X-ray diffraction results of prepared Cu NPs-2 and Cu/CuCN-2.

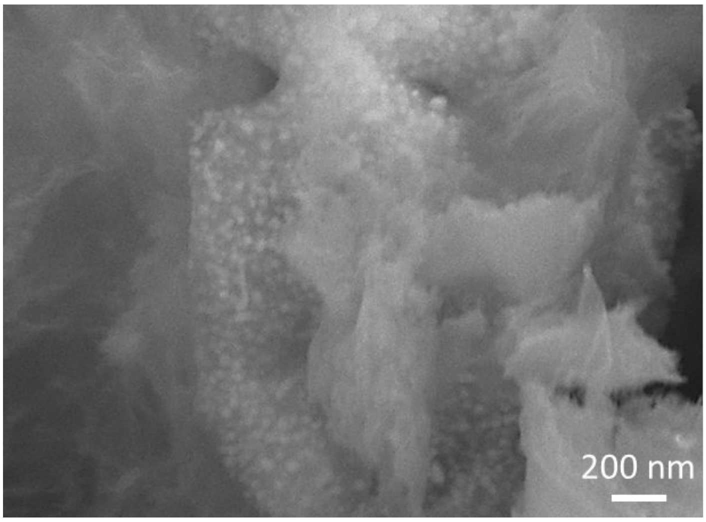

Figure S10. SEM results of prepared Cu/CuCN catalyst.

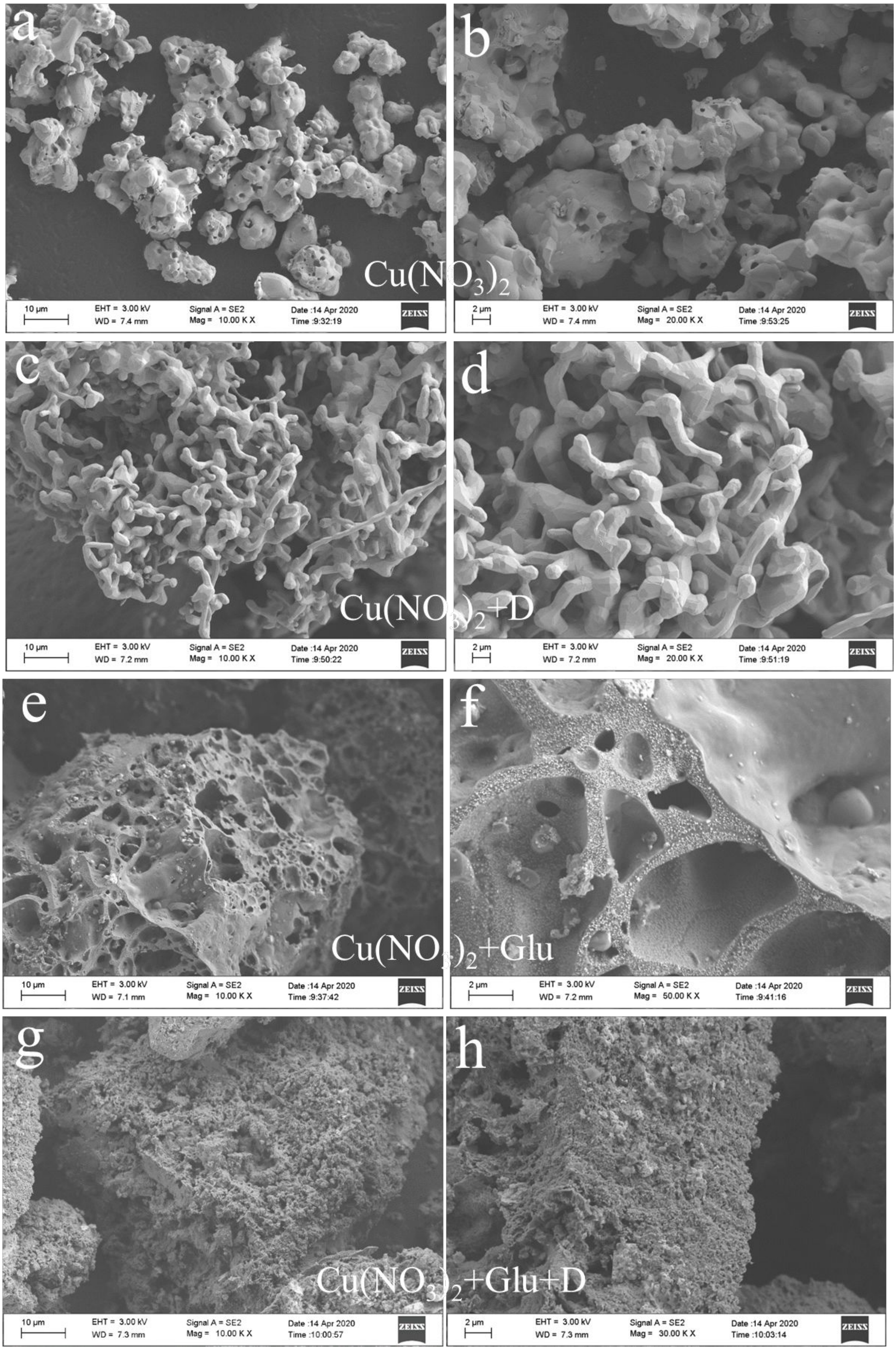

Figure S11. SEM results of obtained $Cu(NO_3)_2$ (a,b), $Cu(NO_3)_2$+D (c,d), $Cu(NO_3)_2$+Glu (e,f), and $Cu(NO_3)_2$+Glu+D (g,h) after 800 ℃ calcination, respectively.

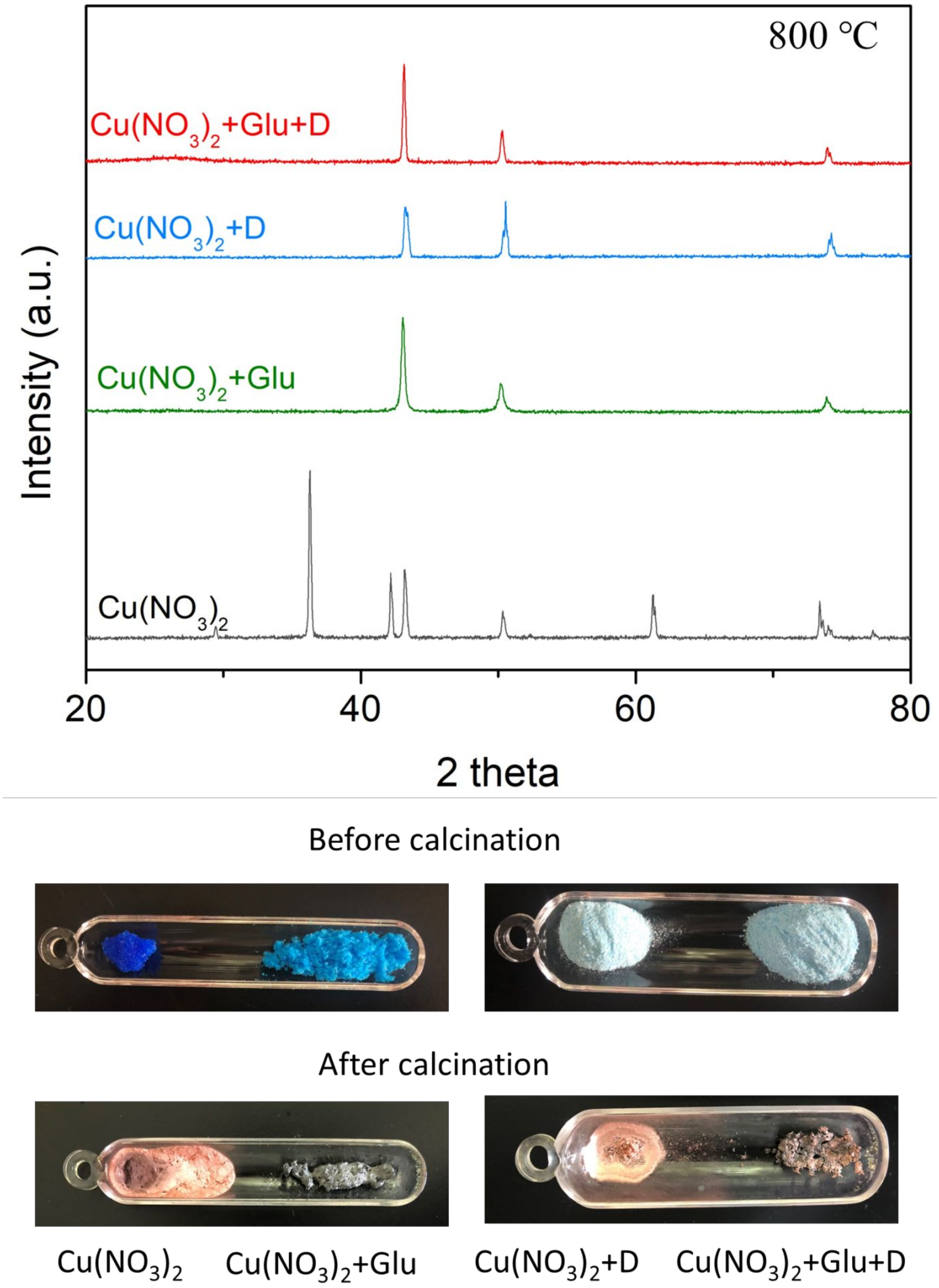

Figure S12. PXRD patterns and photograph of $Cu(NO_3)_2$, $Cu(NO_3)_2$+D, $Cu(NO_3)_2$+Glu and $Cu(NO_3)_2$+Glu+D after 800 ℃ calcination, respectively.

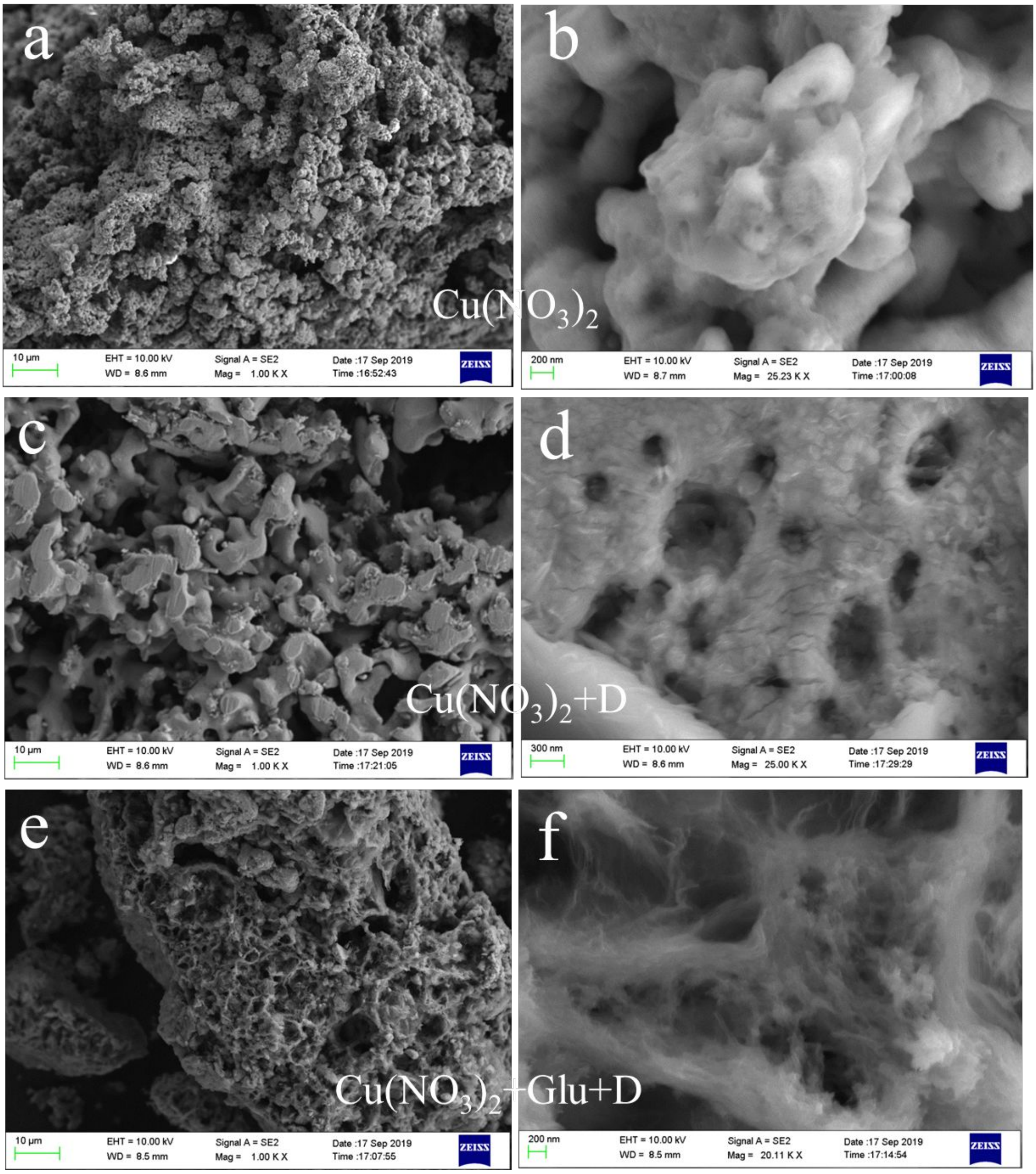

Figure S13. SEM results of (a, b) $Cu(NO_3)_2$, (c, d) $Cu(NO_3)_2$+D, (e, f) $Cu(NO_3)_2$+Glu+D after 700 °C calcination, respectively.

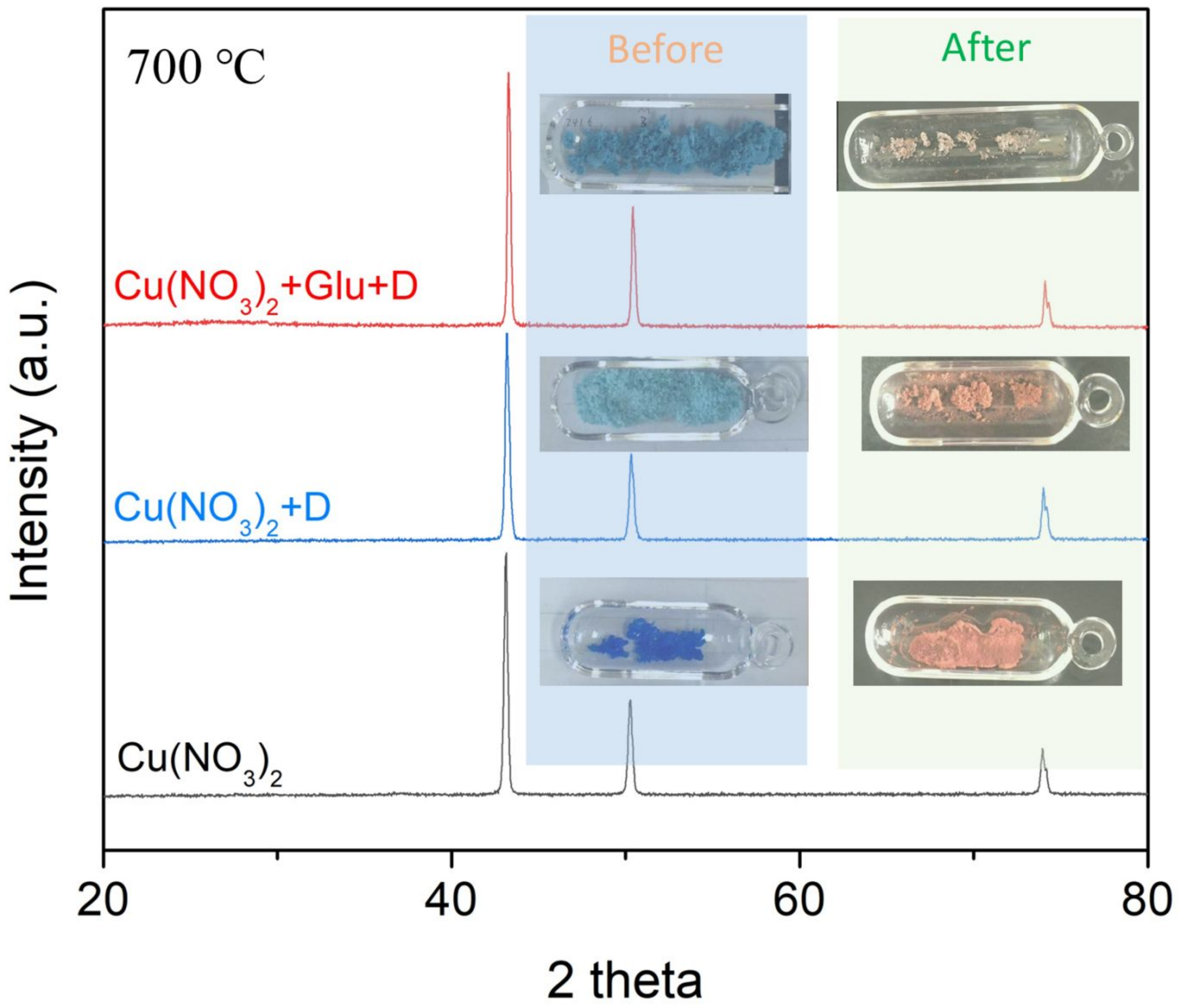

Figure S14. PXRD patterns of $Cu(NO_3)_2$, $Cu(NO_3)_2$+D, $Cu(NO_3)_2$+Glu+D after 700 ℃ calcination, respectively.

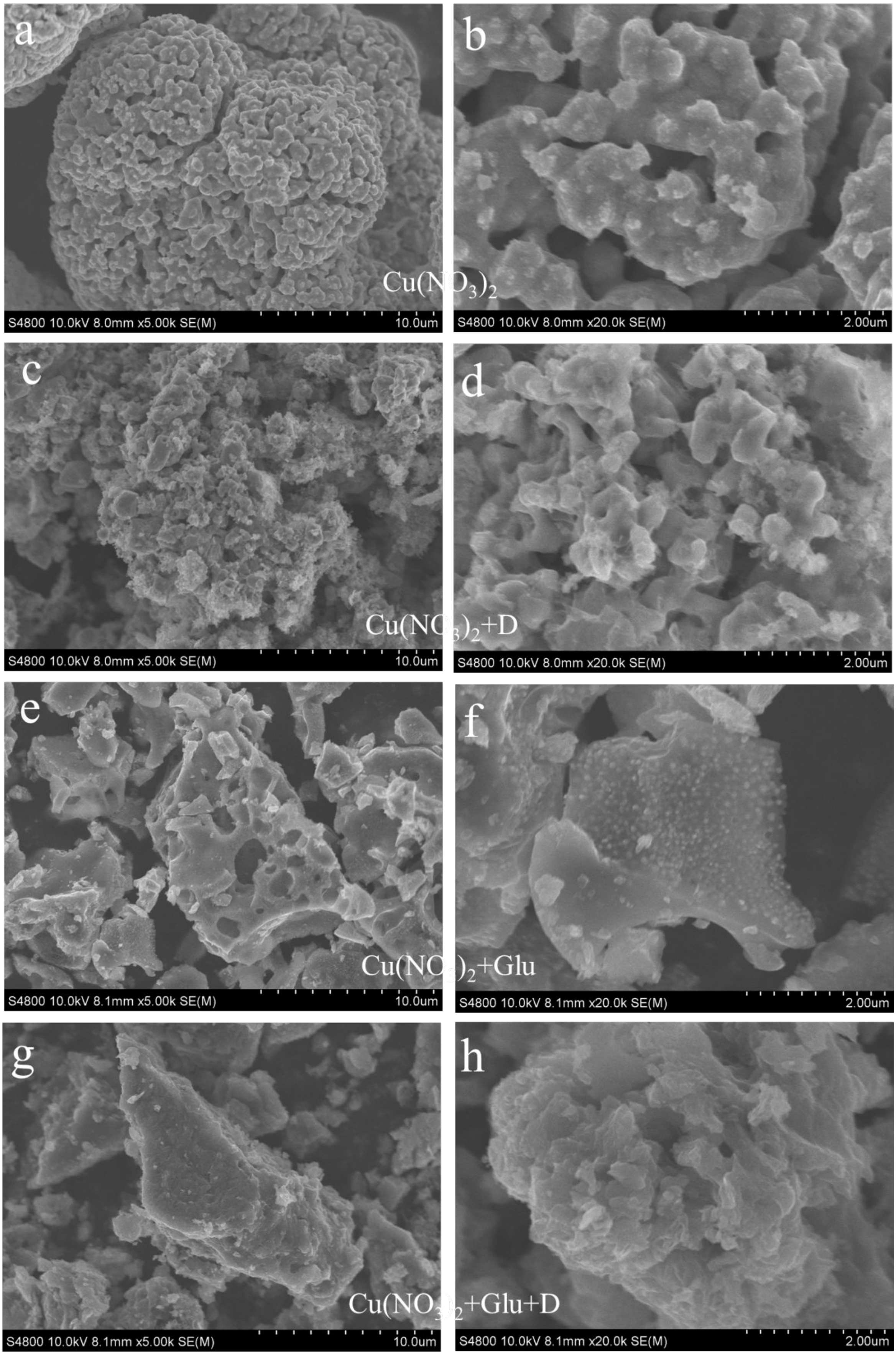

Figure S15. SEM results of (a, b) $Cu(NO_3)_2$, (c, d) $Cu(NO_3)_2$+D, (e, f) $Cu(NO_3)_2$+Glu, and (g, h) $Cu(NO_3)_2$+Glu+D after 600 ℃ calcination, respectively.

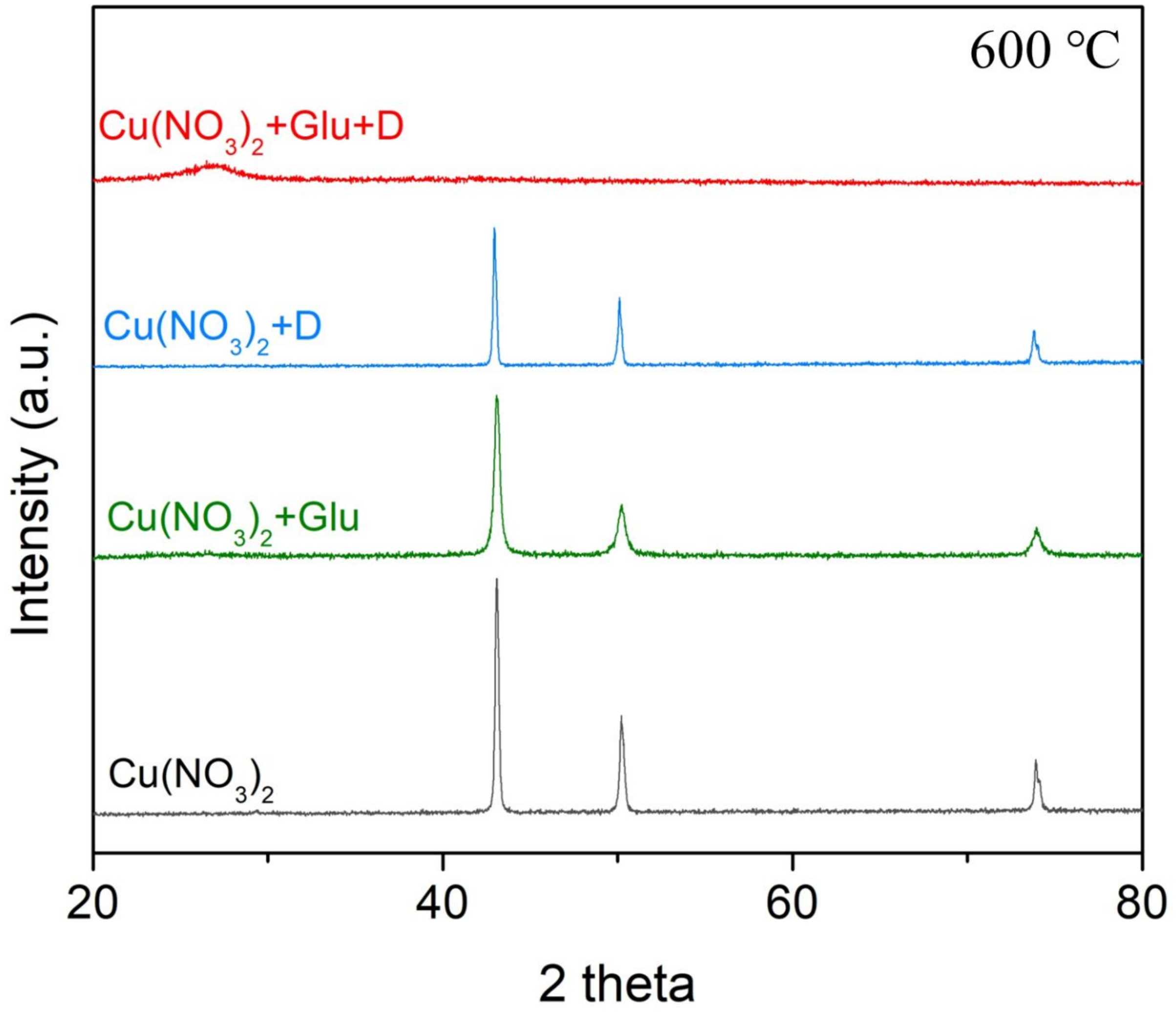

Figure S16. PXRD patterns of $Cu(NO_3)_2$, $Cu(NO_3)_2$+D, $Cu(NO_3)_2$+Glu and $Cu(NO_3)_2$+Glu+D after 600 ℃ calcination, respectively.

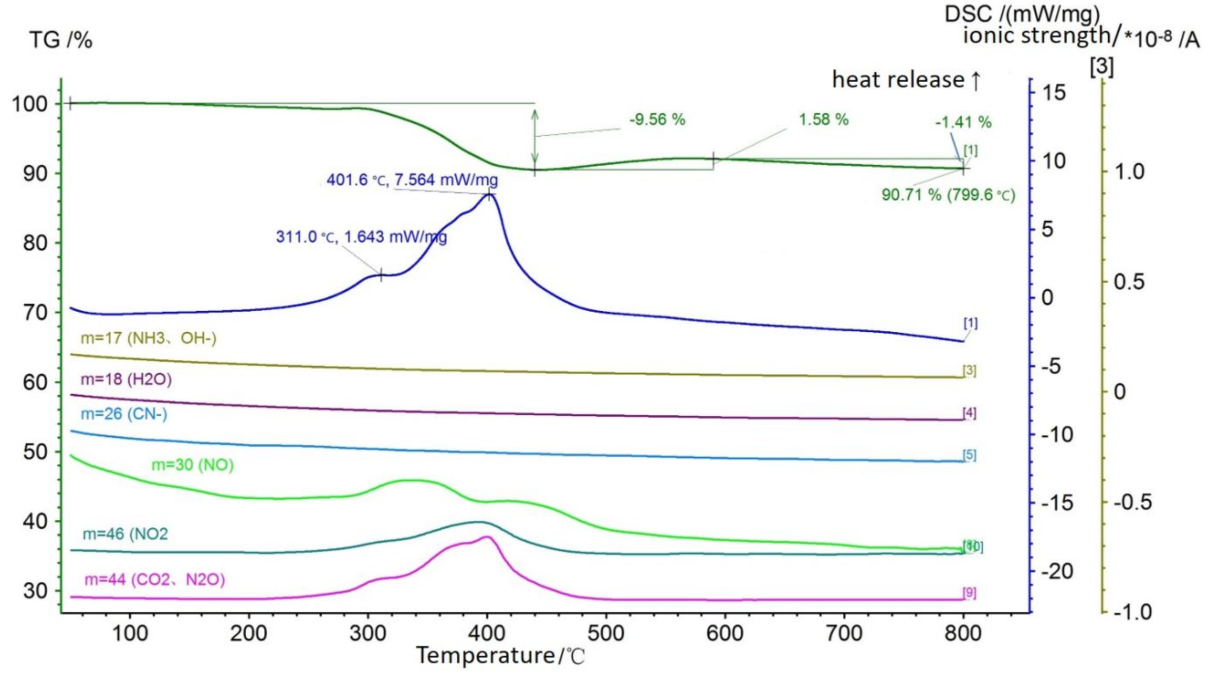

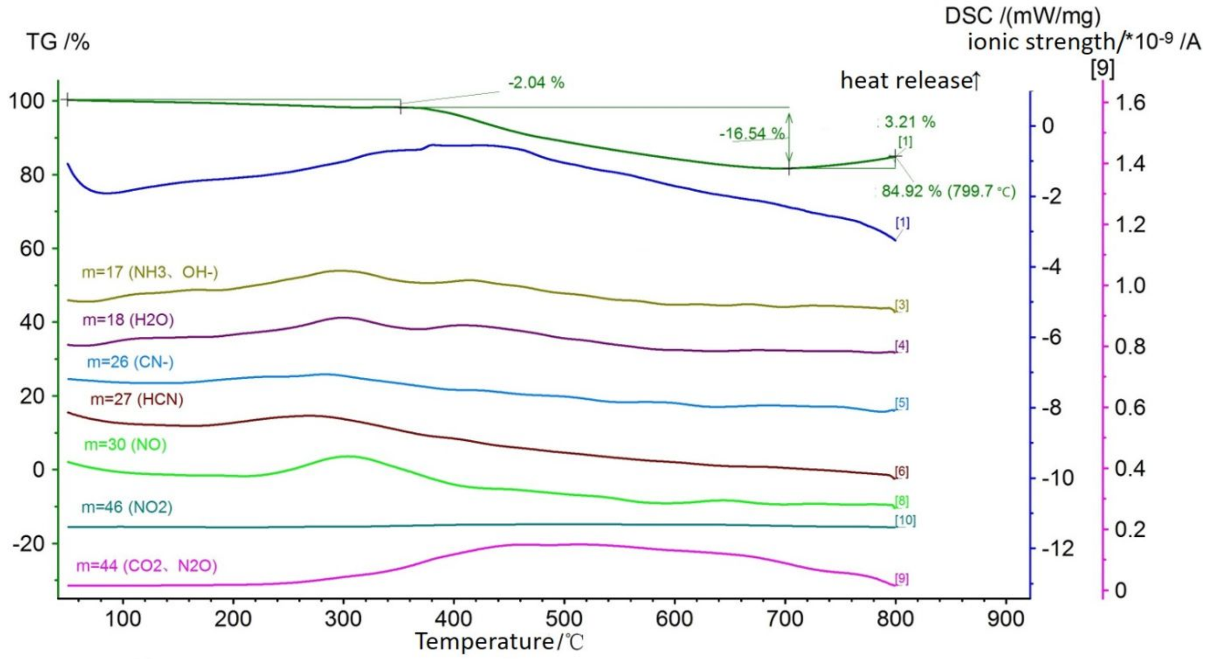

Figure S17. Thermal Gravimetric-mass spectra of Cu NPs (up) and Cu/CuCN (down).

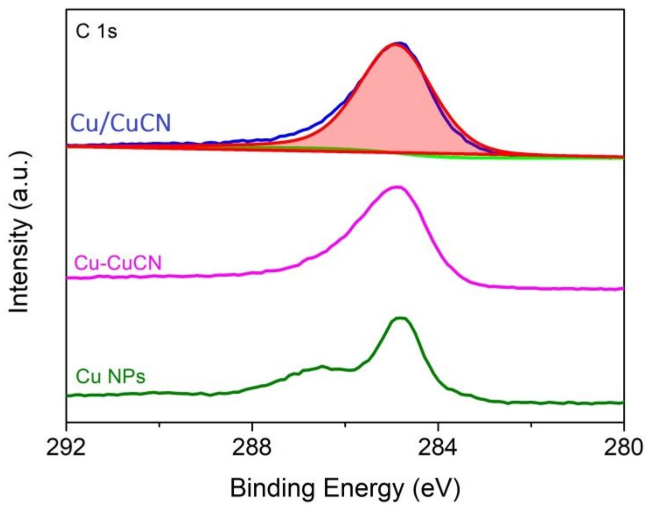

Figure S18. The high resolution XPS spectra of C 1s from obtained three samples.

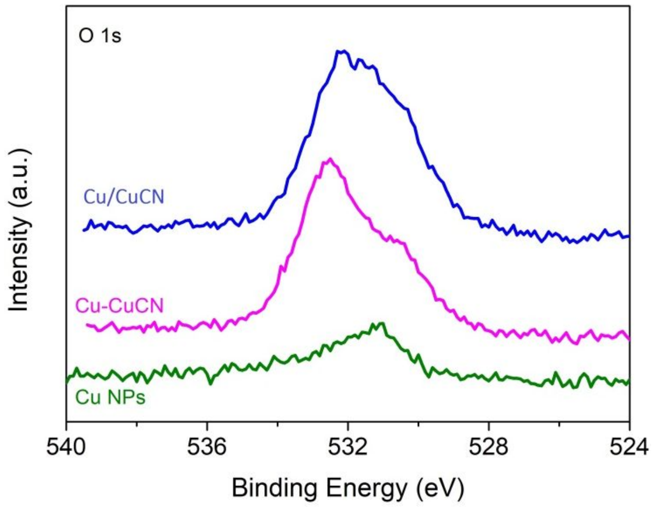

Figure S19. The XPS spectra of O 1s from the Cu/CuCN, Cu-CuCN and Cu NPs samples.

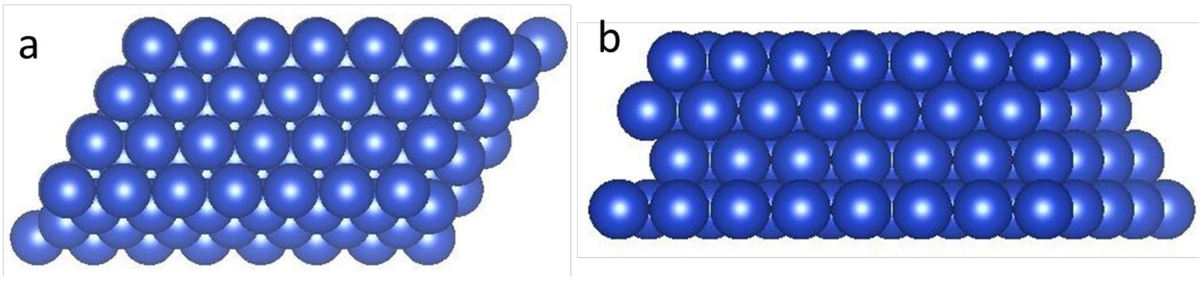

Figure S20. The Schematic structures of on a Cu(111) facet (a and b).

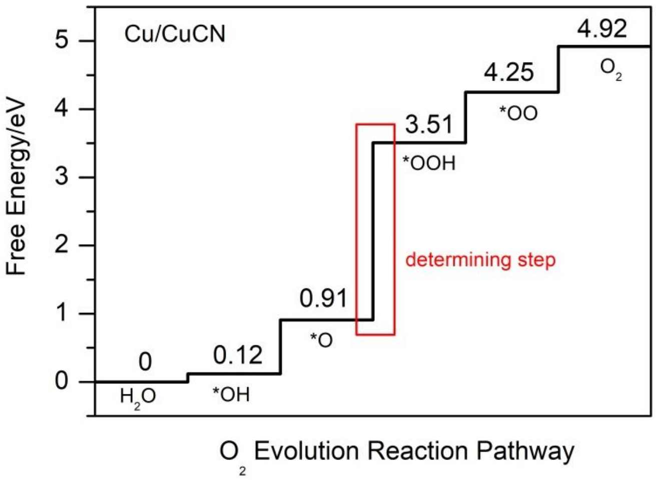

Figure S21. The calculated OER free energy diagram of the Cu/CuCN catalysts under alkaline conditions.

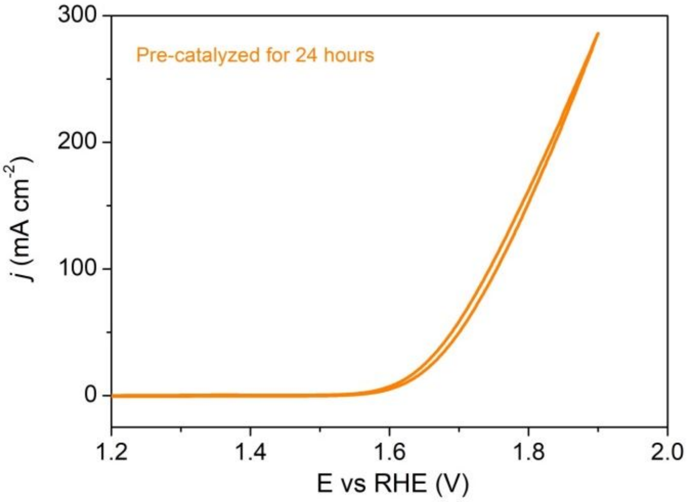

Figure S22. The cyclic voltammetry (CV) was employed to determine "onset potential".

For example, the overpotentials for water oxidation were about 320 mV on the beginning of a rapid increase of currents distinguished from the nearly horizontal line, so we used 1.55 V vs. RHE (1.23 V+0.32 V) as the "onset potential". At the same time, the results of CV also demonstrated that the onset potential was around 1.55 V with intersection of the returned CV curve and the horizontal line at 0 mA cm^{-2}.

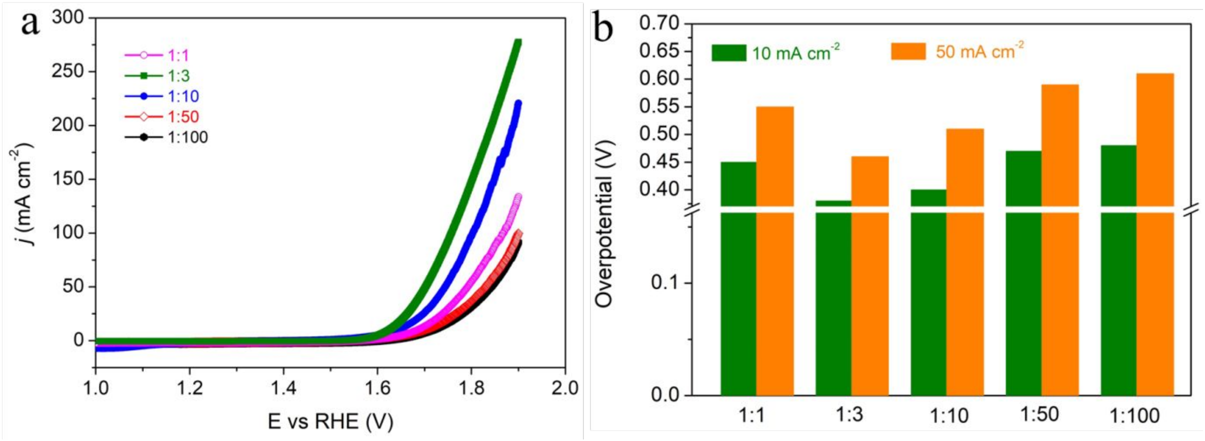

Figure S23. OER polarization curves of the obtained catalysts. (a) The CVs of Cu/CuCN from various ratios of CuGT to D (dicyandiamide): 1:1, 1:3, 1:10, 1:50, and 1:100. (b) The comparison of needing overpotentials at 10 and 50 mA cm^{-2}.

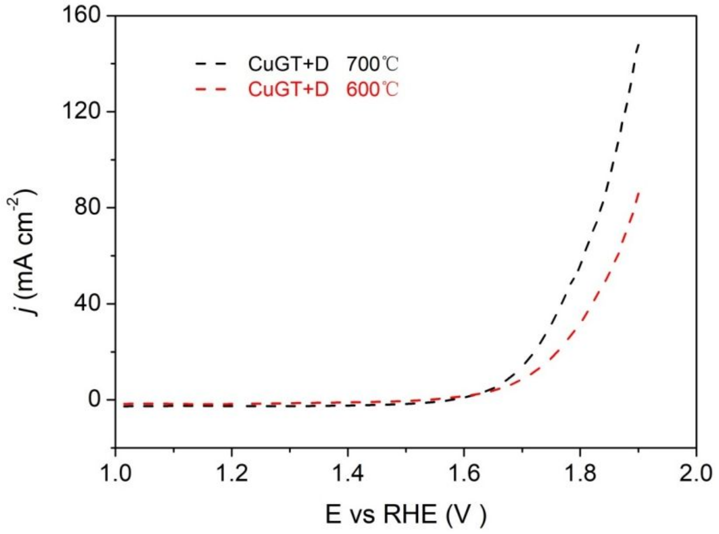

Figure S24. The OER polarization curves of Cu/CuCN-1 and Cu/CuCN-2 catalysts from CuGT+D after 700 and 600 ℃ calcination, respectively.

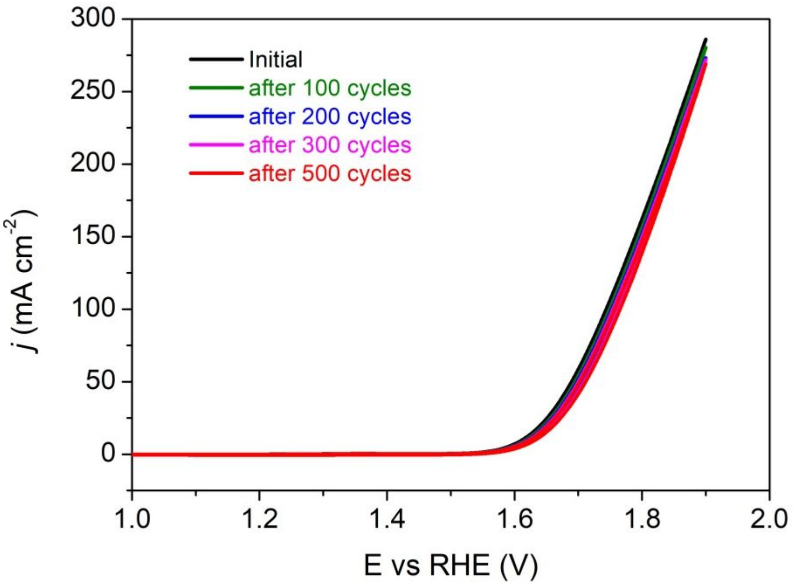

Figure S25. The CVs of Cu/CuCN after different cycles. The results demonstrated that Cu/CuCN had better long-term stability.

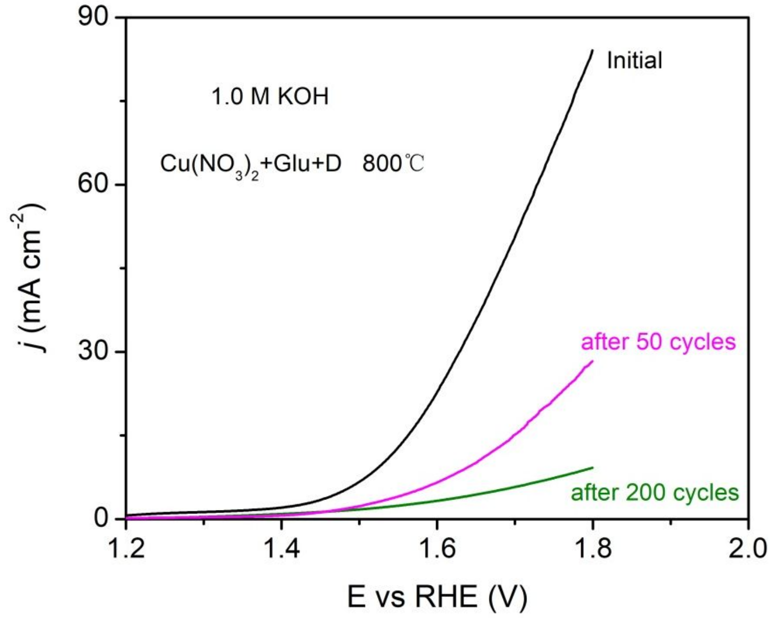

Figure S26. The LSV of $Cu(NO_3)_2$+Glu+D after 800 ℃ calcination in 1.0 M KOH solution. The results demonstrated that the catalysts from $Cu(NO_3)_2$+Glu+D after 800 ℃ calcination had a significantly declining electrocatalytic performance and poor stability.

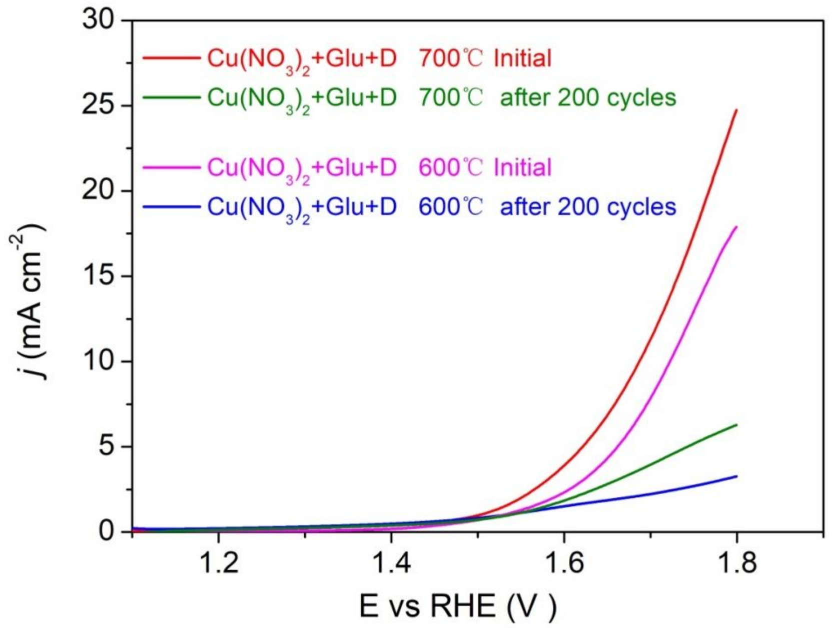

Figure S27. The OER polarization curves of catalysts from $Cu(NO_3)_2$+Glu+D after 600 and 700 ℃ calcination.

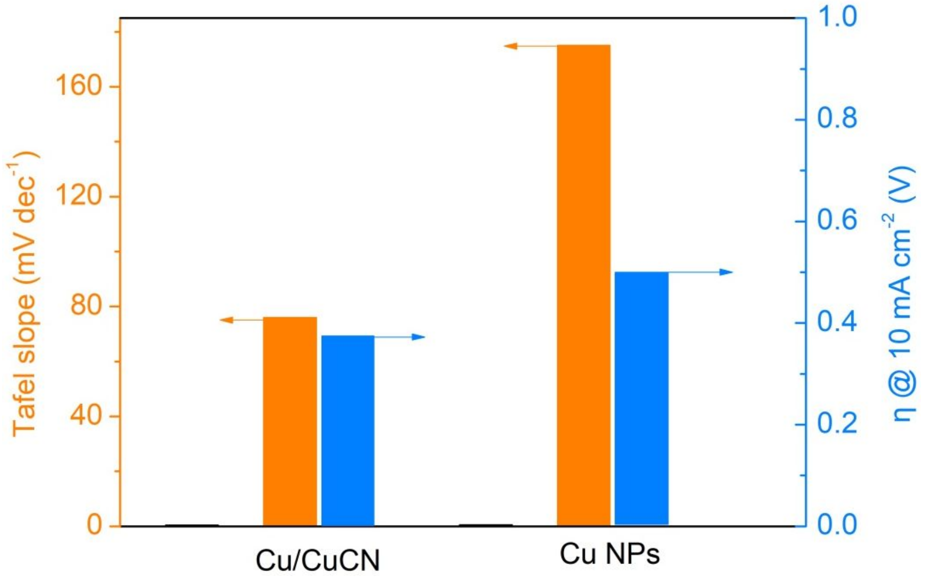

Figure S28. Detail comparison including Tafel slope (left axis) and overpotential at 10 mA cm^{-2} (right axis).

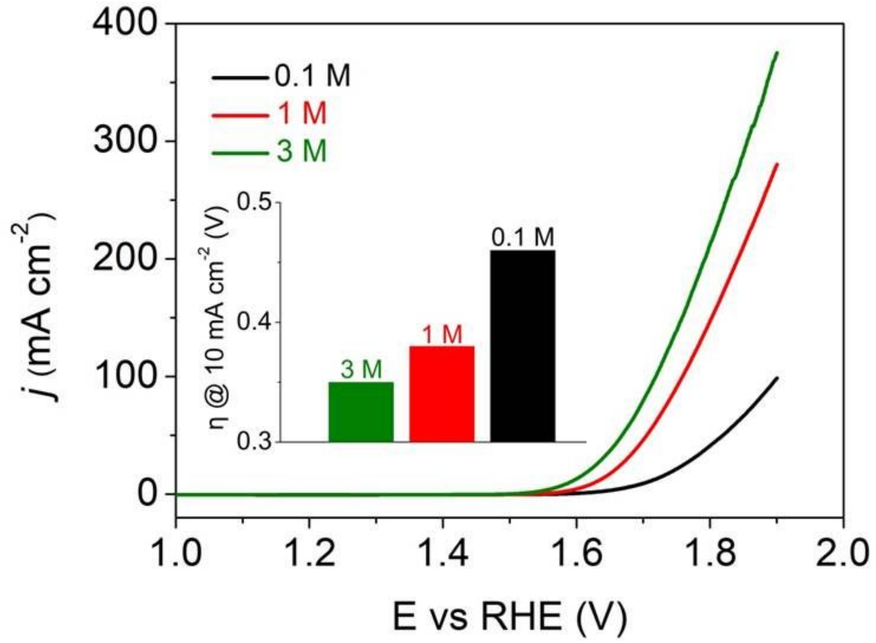

Figure S29. Water oxidation curves of the Cu/CuCN electrode in 0.1, 1.0 and 3.0 M KOH solutions.

The OER processes of the Cu/CuCN electrode in 0.1, 1.0 and 3.0 M KOH exhibited the different onset potential of 1.60, 1.55 and 1.50 V, respectively, as displayed in Figure S29. The more rapid increase in current was obviously observed for 3.0 M KOH under higher potentials, which attributed to higher conductivity of the electrolyte. At the same current density (j) = 100 mA cm^{-2}, η = 480 mV was obtained in 3.0 M KOH solution, while 530 mV and 670 mV is obtained in 1.0 and 0.1 M KOH, respectively.

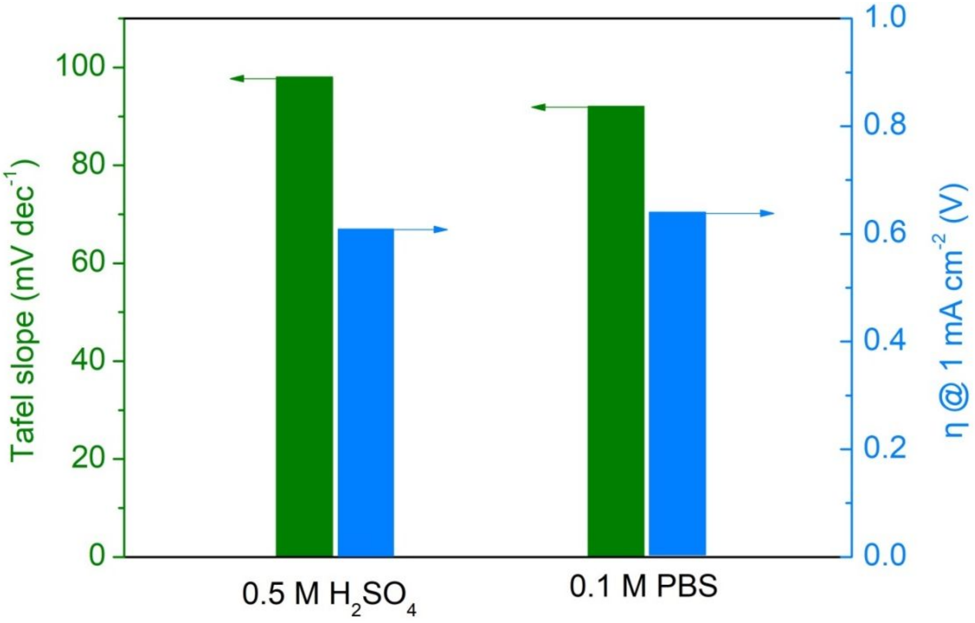

Figure S30. The Tafel slope (left axis) and overpotential at 1 mA cm^{-2} (right axis) under 0.5 M H_2SO_4 and 0.1 M PBS solutions.

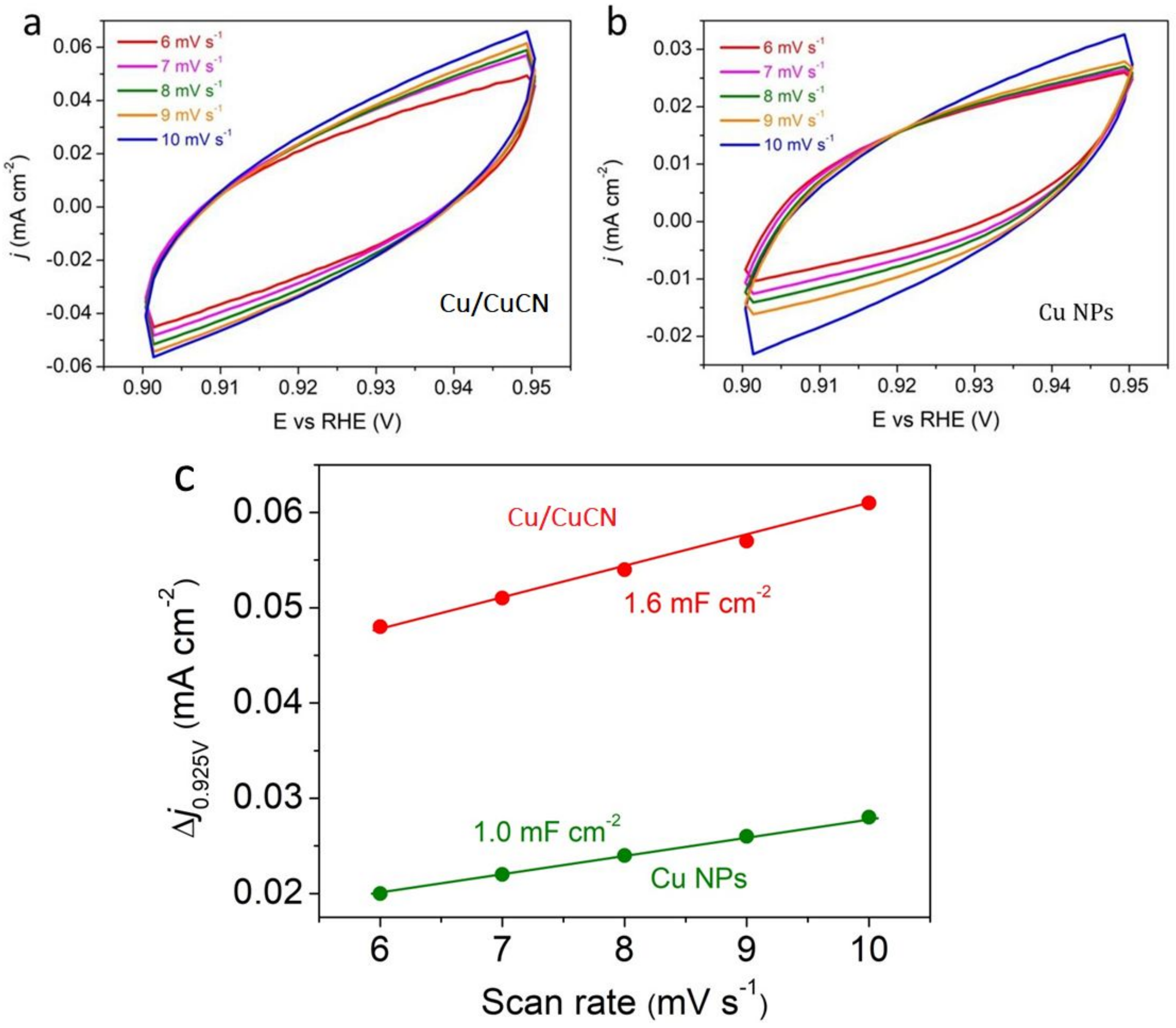

Figure S31. CV curves of the prepared catalysts at non-Faraday area. The CV curves were tested in 1.0 M KOH at potential range without chemical reaction under scan rates of 6, 7, 8, 9 and 10 mV s^{-1}. The CV curves of Cu/CuCN (a) and Cu NPs (b). (c) The fitting plots showing C_{dl} for OER.

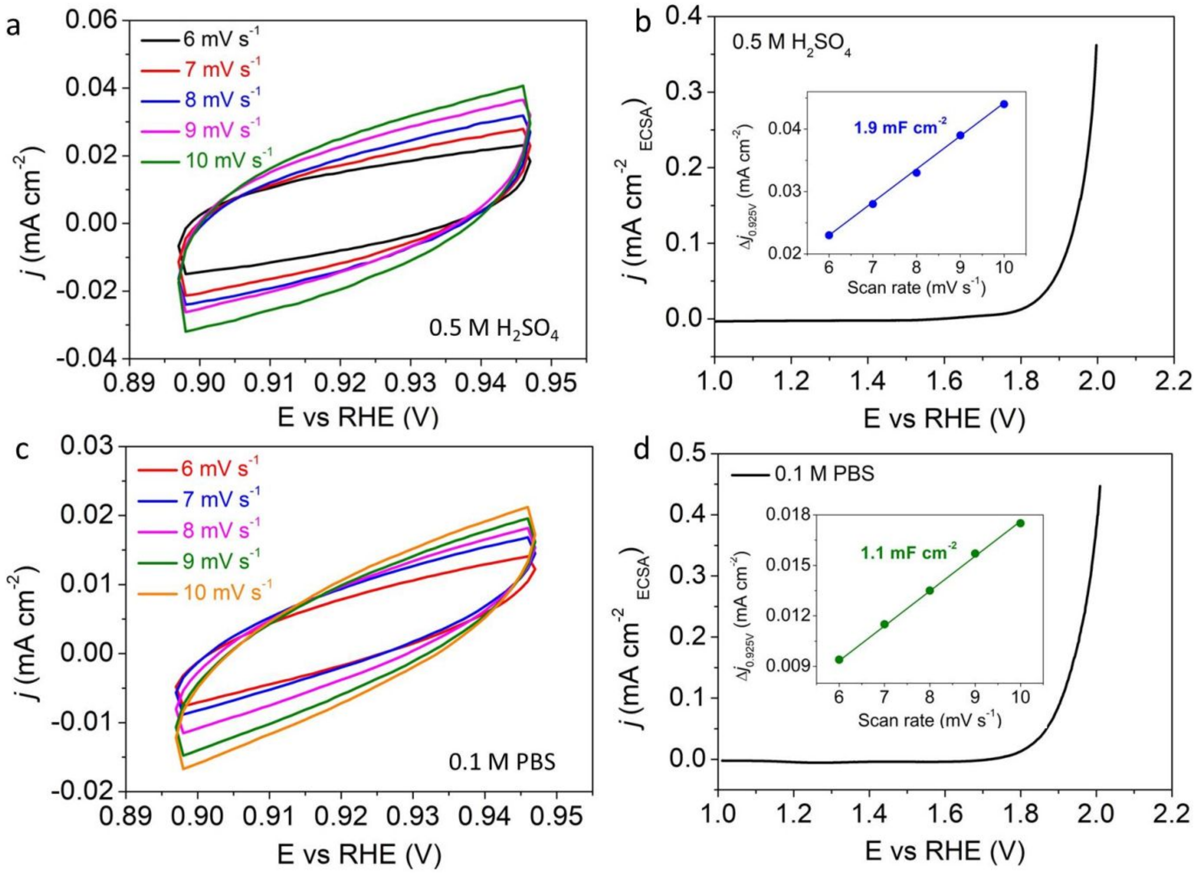

Figure S32. The obtained current density versus scan rate to assess the C_{dl} of the Cu/CuCN catalysts in (a) 0.5 M H_2SO_4 and (c) 0.1 M PBS solution. (b) and (d) Polarization curves for OER under 0.5 M H_2SO_4 and 0.1 M PBS solutions on Cu/CuCN by normalizing to the ECSA, respectively. The Inset of (b) and (d) represents the values of C_{dl}.

Our experiments revealed a slightly different C_{dl} of Cu/CuCN in 0.5 M H_2SO_4 and 0.1 M PBS solution by CVs at different scan rate. As shown in Inset of Figure S32b and d, the C_{dl} were 1.9 mF cm^{-2} (0.5 M H_2SO_4), and 1.1 mF cm^{-2} (0.1 M PBS), respectively. Correspondingly, the ECSAs determined for Cu/CuCN under 0.5 M H_2SO_4 and 0.1 M PBS (pH 7) environments were 31.7 cm^2 and 18.3 cm^2, respectively. OER polarization curves were normalized by ECSA to evaluate the actual activity of the resulting catalysts, as shown in Figure S32b and d.

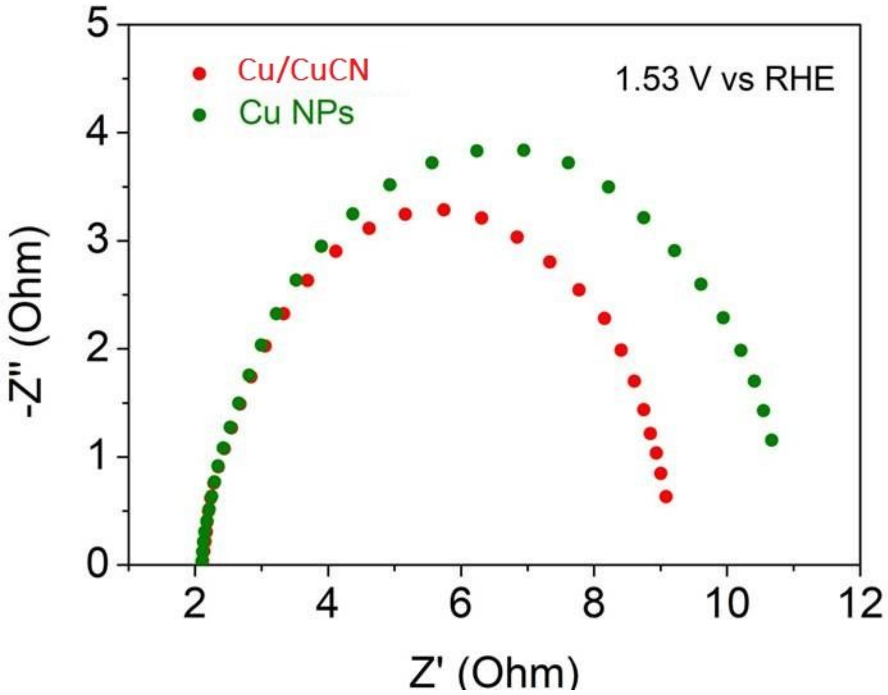

Figure S33. AC impedance of Cu/CuCN and Cu NPs catalysts.

As we all know, the impedance at the high-frequency limit is defined as its Ohmic resistance (R_s), and the diameter of the semicircle is its charge-transfer resistance (R_{ct}). In other words, the equivalent circuit of electrochemical impedance spectroscopy (EIS) is consisted of the R_s, R_{ct}, and a constant phase element (CPE). Generally, a smaller R_{ct} value indicates a faster electron transfer. Furthermore, we conducted EIS measurements on the as-prepared catalysts by Nyquist plots.

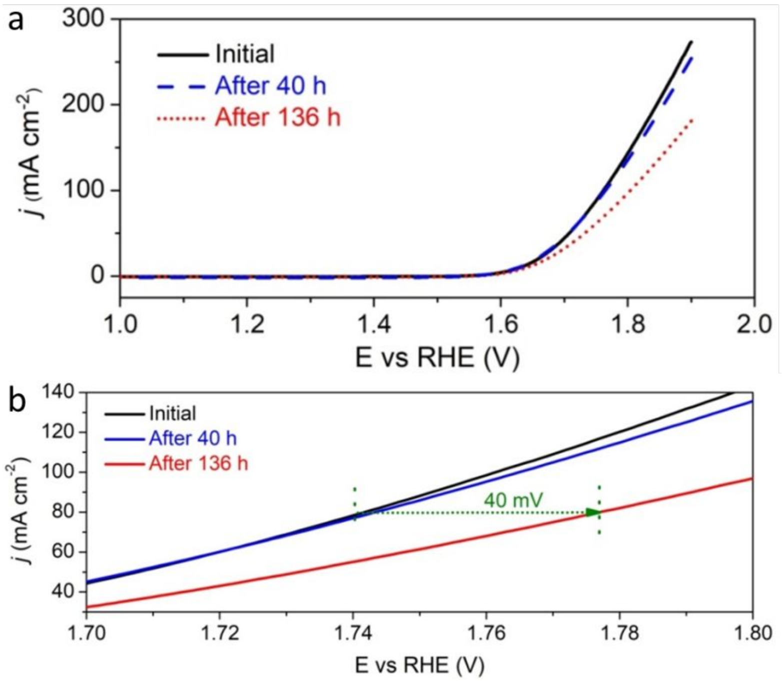

Figure S34. The polarization curves of the Cu/CuCN electrode before and after the stability test in 1.0 M KOH (a-b).

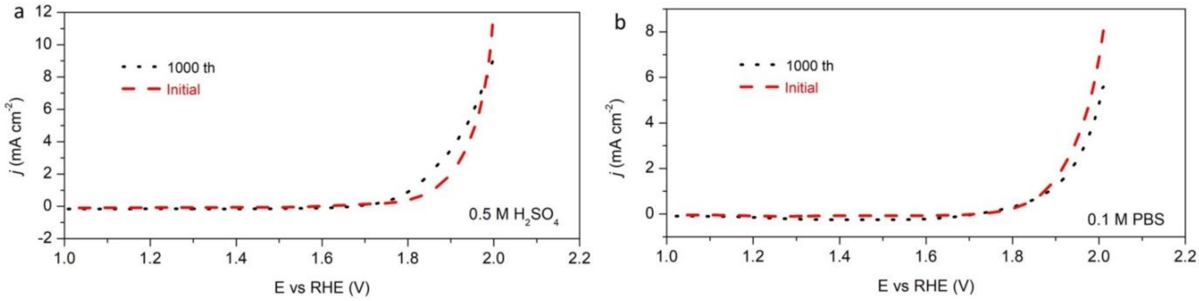

Figure S35. Electrochemical stability of the Cu/CuCN electrode under (a) acidic and (b) neutral conditions.

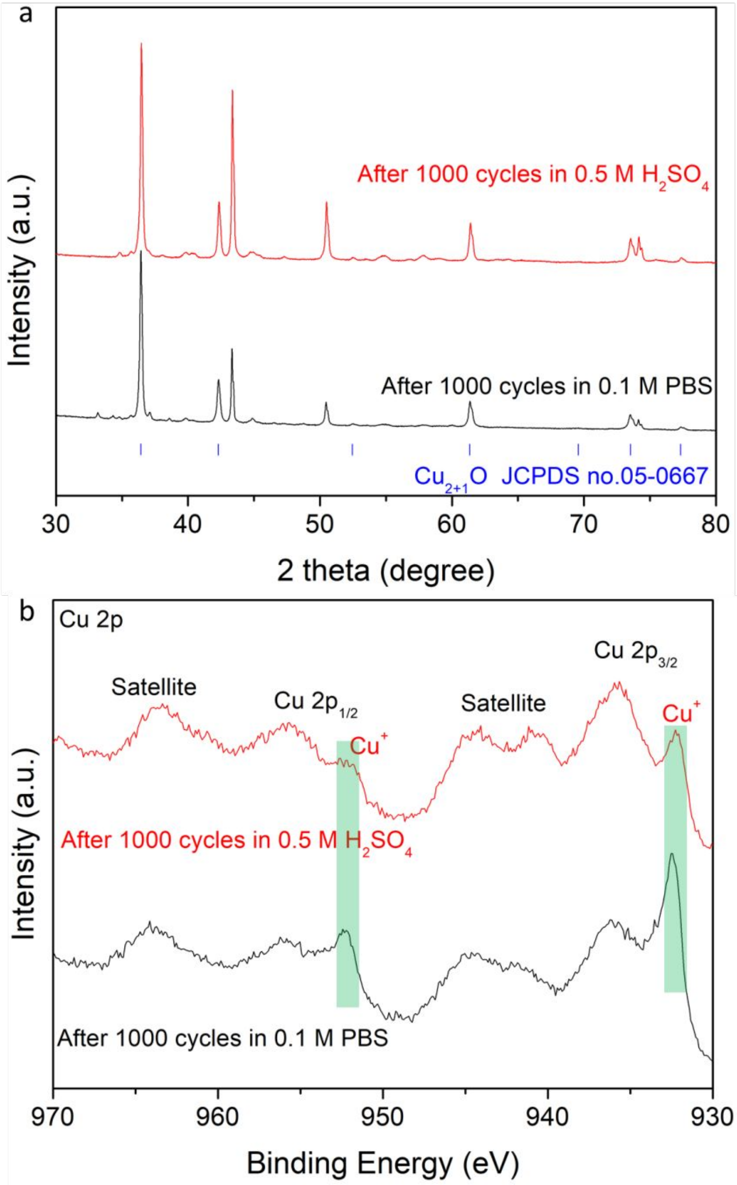

Figure S36. Postcharacterizations of the Cu/CuCN samples after 1000 cycles OER. (a) PXRD pattern, (b) High-resolution XPS spectra of Cu 2p.

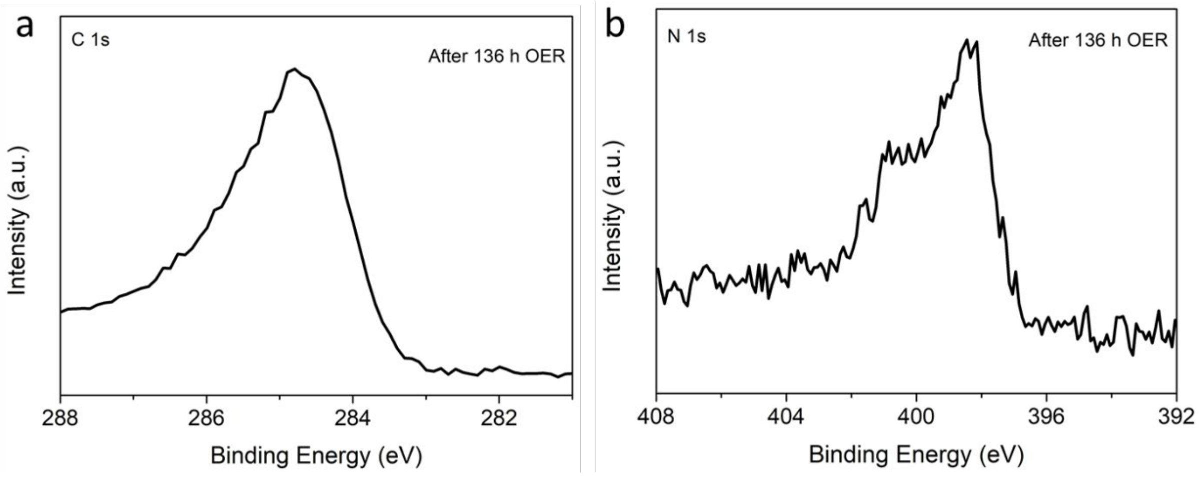

Figure S37. The XPS spectra of C 1s (a), and N 1s (b) from the Cu/CuCN after 136 h OER.

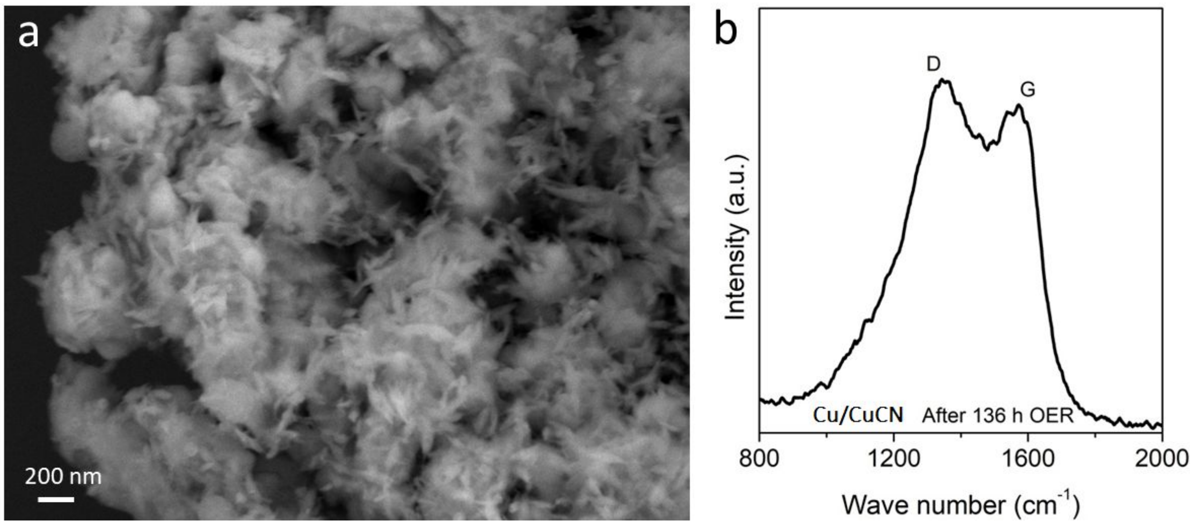

Figure S38. The (a) SEM and (b) Raman spectra from the Cu/CuCN after 136 h OER.

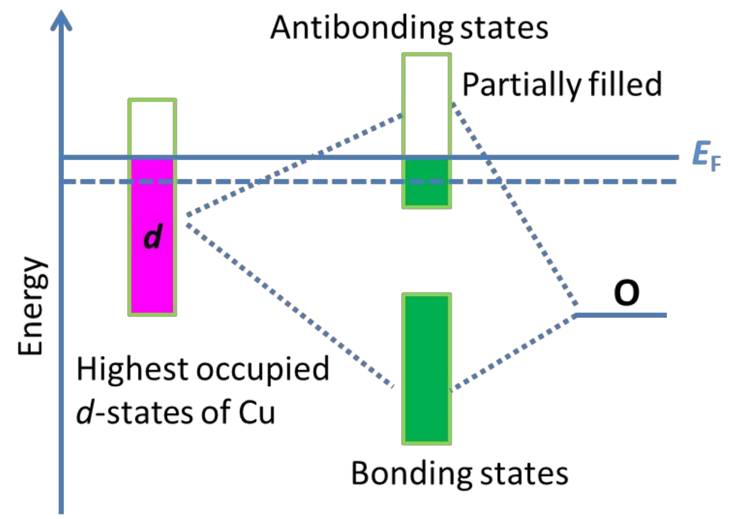

Figure S39. Schematic illustration of orbital hybridization of Cu *3d* and O *2p*. The colored parts represent the electron filling.

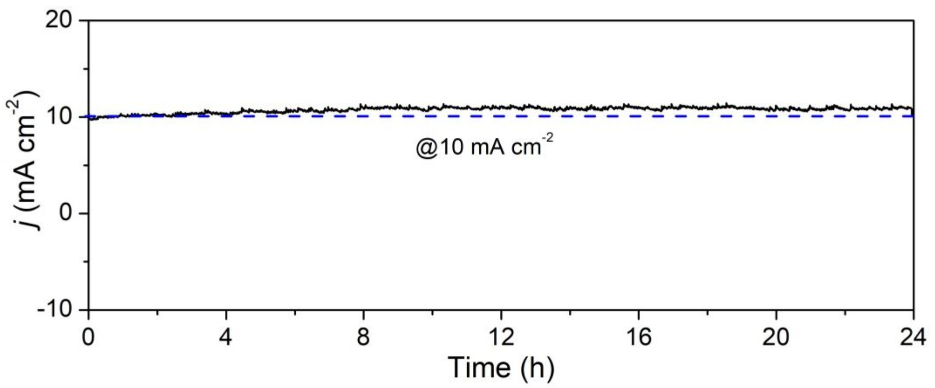

Figure S40. I-t curves of Cu/CuCN||$MoNi_4/MoO_{3-x}$ coupled water electrolysis cell at 10 mA cm^{-2} in 1.0 M KOH solution.

Table S1. The atomic ratio of different elements of Cu/CuCN.

Element	*Atom percent*
Cu	*24.30*
C	*31.24*
N	*0.94*
O	*43.51*

Table S2. OER performances of Cu- and other transition metals–based OER electrocatalysts.

Catalysts	*J* (mA cm^{-2})	*η* (mV)	Tafel (mV dec^{-1})	Electrolyte	References
Cu/CuCN	10	250	97	1.0 M KOH	This work
		380	76		
Cu NPs	10	500	175	1.0 M KOH	This work
Cu(TCNQ)NA/CF	20	380	161	1.0 M KOH	[1]
Cu@NCNT/Co*x*O*y*	10	370	/	0.1 M KOH	[2]
Cu@NCNT	8	470	/	0.1 M KOH	
Cu–N–C NA/CF	20	314	115	1.0 M KOH	[1]
Pt-Cu@PCN920	10	350	105	1.0 M KOH	[3]
$Cu(OH)_2$/CM_100CVs	10	484	165	1.0 M KOH	[4]
CuO/CM-AN_100CVs	10	441	143	1.0 M KOH	
Cu_2S/CM_200CVs	10	380	96	1.0 M KOH	
$CuCo_2S_4$,	10	310	86	1.0 M KOH	[5]
$Cu_{0.5}Co_{2.5}S_4$	10	370	98	1.0 M KOH	
Electrode 1	10	313	162±0.7	0.1 M KOH	[6]
Electrode 2	10	420	187±3.0	0.1 M KOH	
Electrode 3	10	480	144±1.0	0.1 M KOH	
Electrode 4	10	470	123±0.4	0.1 M KOH	
AN-CuNiFe	10	224	44	1.0 M KOH	[7]
CuNiS NWs	30	307	81	1.0 M KOH	[8]
CuNi NWs	30	432	125	1.0 M KOH	
nanostructured Cu oxide (1)	10	290	64	1.0 M NaOH	[9]
nanostructured Cu oxide (2)	10	400	80	1.0 M NaOH	
nanostructured Cu oxide (3)	10	450	89	1.0 M NaOH	
Cu/$Cu(OH)_2$-CuO nanorods	10	417	76	0.1 M KOH	[10]
3D $Cu(OH)_2$ -NWAs/Cu foil	10	530	86	0.1 M NaOH	[11]
$Fe(OH)_3$:$Cu(OH)_2$ Core–Shell	10	365	42	1.0 M KOH	[12]
$Cu(OH)_2$/CF	10	400	73	1.0 M KOH	
Cu bifunctional	1	749	/	0.1 M KBi	[13]
CuO from Cu–EA	10	475	/	1.0 M KOH	[14]
Cu@CuO–C-0	10	340	156	1.0 M KOH	[15]
Cu@Cu_2O–C	10	390	165	1.0 M KOH	
Cu–C	10	414	204	1.0 M KOH	
CuO–C	10	456	218	1.0 M KOH	
Cu nanoparticles	10	480	/	0.5 M KOH	[16]

Cu_3P@NF	10	320	54	1.0 M KOH	[17]
Annealed CuO-3	10	580	/	1.0 M KOH	[18]
Cu–B_i	1	530	/	pH 9 (borate buffer)	[19]
Cu-N/graphene	5	660	/	0.1 M KOH	[20]
$Cu_{2-x}Se/Ni_3Se_4$	10	230	48	0.1 M KOH	[21]
Cu_3P microsheets	10	290	77	1.0 M KOH	[22]
Exfoliated NiFe LDHs	10	300	40	1.0 M KOH	[23]
NiFeV LDHs	10	192	42	1.0 M KOH	[24]
Co_3O_4	10	290	84	1.0 M KOH	[25]
Free-Standing Holey $Ni(OH)_2$	10	293	65	1.0 M KOH	[26]
Ni_2P-VP_2/NF	50	306	49	1.0 M KOH	[27]
$Ni_2P_4O_{12}$/CC	10	280	156	1.0 M KOH	[28]
FeCoW alloy	10	232	/	1.0 M KOH	[29]
MoS_2-Ni_3S_2 HNRs/NF	10	249	66	1.0 M KOH	[30]
IrO_2/NF	10	285	46	1.0 M KOH	[31]

Table S3. Comparison of the overall water splitting activities among different non-precious metal electrocatalysts tested under alkaline conditions (1 M KOH).

Catalysts	Electrolyte	Potential (V) @ 10 mA cm^{-2}	References
$Cu/CuCN//MoNi_4/MoO_{3-x}$	1.0 M KOH	1.53	this work
Porous MoO_2//Porous MoO_2	1.0 M KOH	1.53	32
NiSe nanowire film/Ni foam	1.0 M KOH	1.63	33
Co-P films/Cu foil	1.0 M KOH	1.64	34
NiO//NiO	1.0 M KOH	1.84	35
NiFe LDH-NS@DG10	1.0 M KOH	1.44	36
$(Ni_{0.33}Fe_{0.67})_2P$	1.0 M KOH	1.49	37
Compact MoO_2/Ni foam	1.0 M KOH	1.73	32
$NiO//Ni_2P$	1.0 M KOH	1.65	35
$NiCo_2O_4//NiCo_2O_4$	1.0 M KOH	1.65	38
$Ni_3S_2//Ni_3S_2$	1.0 M KOH	1.76<br>(13 mA cm^2)	39
NiFe LDH//NiO/Ni-CNT	1.0 M KOH	1.42	40
$CoS_{0.46}P_{0.54}//CoS_{0.46}P_{0.54}$	1.0 M KOH	1.62	41
$Mo\text{-}NiCo_2O_4//Co_{5.47}N/NF$	1.0 M KOH	1.56	42

References:

(1) Zhu, X.; Shi, X.; Asiri, A. M.; Luo, Y.; Sun, X. Efficient Oxygen Evolution Electrocatalyzed by a Cu Nanoparticle-Embedded N-Doped Carbon Nanowire Array. *Inorg. Chem. Front.* **2018**, *5*, 1188-1192.

(2) Zhao, X.; Li, F.; Wang, R.; Seo, J.-M.; Choi, H.-J.; Jung, S.-M.; Mahmood, J.; Jeon, I.-Y.; Baek, J.-B. Controlled Fabrication of Hierarchically Structured Nitrogen-Doped Carbon Nanotubes as a Highly Active Bifunctional Oxygen Electrocatalyst. *Adv. Funct. Mater.* **2017**, *27*, 1605717.

(3) Nadeem, M.; Yasin, G.; Bhatti, M. H.; Mehmood, M.; Arif, M.; Dai, L. Pt-M Bimetallic Nanoparticles (M = Ni, Cu, Er) Supported on Metal Organic Framework-Derived N-Doped Nanostructured Carbon for Hydrogen Evolution and Oxygen Evolution Reaction. *J. Power Sources* **2018**, *402*, 34-42.

(4) Zuo, Y.; Liu, Y.; Li, J.; Du, R.; Han, X.; Zhang, T.; Arbiol, J.; Divins, N. J.; Llorca, J.; Guijarro, N.; Sivula, K.; Cabot, A. In Situ Electrochemical Oxidation of Cu_2S into CuO Nanowires as a Durable and Efficient Electrocatalyst for Oxygen Evolution Reaction. *Chem. Mater.* **2019**, *31*, 7732-7743.

(5) Chauhan, M.; Reddy, K. P.; Gopinath, C. S.; Deka, S. Copper Cobalt Sulfide Nanosheets Realizing a Promising Electrocatalytic Oxygen Evolution Reaction. *ACS Catal.* **2017**, *7*, 5871-5879.

(6) Gao, D.; Liu, R.; Biskupek, J.; Kaiser, U.; Song, Y.-F.; Streb, C. Modular Design of Noble-Metal-Free Mixed Metal Oxide Electrocatalysts for Complete Water Splitting. *Angew. Chem., Int. Ed.* **2019**, *58*, 4644-4648.

(7) Cai, Z.; Li, L.; Zhang, Y.; Yang, Z.; Yang, J.; Guo, Y.; Guo, L. Amorphous Nanocages of Cu-Ni-Fe Hydr(oxy)oxide Prepared by Photocorrosion for Highly Efficient Oxygen Evolution. *Angew. Chem., Int. Ed.* **2019**, *58*, 4189-4194.

(8) Cao, D.; Cheng, D. One-Pot Synthesis of Copper–Nickel Sulfide Nanowires for Overall Water Splitting in Alkaline Media. *Chem. Commun.* **2019**, *55*, 8154-8157.

(9) Huan, T. N.; Rousse, G.; Zanna, S.; Lucas, I. T.; Xu, X.; Menguy, N.; Mougel, V.; Fontecave, M. A Dendritic Nanostructured Copper Oxide Electrocatalyst for the Oxygen Evolution Reaction. *Angew. Chem., Int. Ed.* **2017**, *56*, 4792-4796.

(10) Cheng, N.; Xue, Y.; Liu, Q.; Tian, J.; Zhang, L.; Asiri, A. M.; Sun, X. Cu/($Cu(OH)_2$-CuO) Core/shell Nanorods Array: In-Situ Growth and Application as an Efficient 3D Oxygen Evolution Anode. *Electrochim. Acta* **2015**, *163*, 102-106.

(11) Hou, C.-C.; Fu, W.-F.; Chen, Y. Self-Supported Cu-Based Nanowire Arrays as Noble-Metal-Free Electrocatalysts for Oxygen Evolution. *ChemSusChem* **2016**, *9*, 2069-2073.

(12) Hou, C.-C.; Wang, C.-J.; Chen, Q.-Q.; Lv, X.-J.; Fu, W.-F.; Chen, Y. Rapid Synthesis of Ultralong $Fe(OH)_3$:$Cu(OH)_2$ Core–Shell Nanowires Self-Supported on Copper Foam as a Highly Efficient 3D Electrode for Water Oxidation. *Chem. Commun.* **2016**, *52*, 14470-14473.

(13) Liu, X.; Zheng, H.; Sun, Z.; Han, A.; Du, P. Earth-Abundant Copper-Based Bifunctional Electrocatalyst for both Catalytic Hydrogen Production and Water Oxidation. *ACS Catal.* **2015**, *5*, 1530-1538.

(14) Liu, X.; Cui, S.; Qian, M.; Sun, Z.; Du, P. In situ Generated Highly Active Copper Oxide Catalysts for the Oxygen Evolution Reaction at Low Overpotential in Alkaline Solutions. *Chem. Commun.* **2016**, *52*, 5546-5549.

(15) Wu, J.-X.; He, C.-T.; Li, G.-R.; Zhang, J.-P. An Inorganic-MOF-Inorganic Approach to Ultrathin CuO Decorated Cu–C Hybrid Nanorod Arrays for an Efficient Oxygen Evolution Reaction. *J. Mater. Chem. A* **2018**, *6*, 19176-19181.

(16) Kumar, B.; Saha, S.; Basu, M.; Ganguli, A. K. Enhanced Hydrogen/oxygen Evolution and Stability of Nanocrystalline (4–6 nm) Copper Particles. *J. Mater. Chem. A* **2013**, *1*, 4728-4735.

(17) Han, A.; Zhang, H.; Yuan, R.; Ji, H.; Du, P. Crystalline Copper Phosphide Nanosheets as an Efficient Janus Catalyst for Overall Water Splitting. *ACS Appl. Mater. Interfaces* **2017**, *9*, 2240-2248.

(18) Liu, X.; Cui, S.; Sun, Z.; Ren, Y.; Zhang, X.; Du, P. Self-Supported Copper Oxide Electrocatalyst for Water Oxidation at Low Overpotential and Confirmation of Its Robustness by Cu K-Edge X-ray Absorption

Spectroscopy. *J. Phys. Chem. C* **2016**, *120*, 831-840.

(19) Yu, F.; Li, F.; Zhang, B.; Li, H.; Sun, L. Efficient Electrocatalytic Water Oxidation by a Copper Oxide Thin Film in Borate Buffer. *ACS Catal.* **2015**, *5*, 627-630.

(20) Wang, J.; Wang, K.; Wang, F.-B.; Xia, X.-H. Bioinspired Copper Catalyst Effective for both Reduction and Evolution of Oxygen. *Nat. Commun.* **2014**, *5*, 5285.

(21) Kim, S.; Mizuno, H.; Saruyama, M.; Sakamoto, M.; Haruta, M.; Kurata, H.; Yamada, T.; Domen, K.; Teranishi, T. Phase Segregated $Cu_{2-x}Se/Ni_3Se_4$ Bimetallic Selenide Nanocrystals Formed through the Cation Exchange Reaction for Active Water Oxidation Precatalysts. *Chem. Sci.* **2020**, *11*, 1523-1530.

(22) Hao, J.; Yang, W.; Huang, Z.; Zhang, C. Superhydrophilic and Superaerophobic Copper Phosphide Microsheets for Efficient Electrocatalytic Hydrogen and Oxygen Evolution. *Adv. Mater. Interfaces* **2016**, *3*, 1600236.

(23) Song, F.; Hu, X. Exfoliation of Layered Double Hydroxides for Enhanced Oxygen Evolution Catalysis. *Nat. Commun.* **2014**, *5*, 4477.

(24) Li, P.; Duan, X.; Kuang, Y.; Li, Y.; Zhang, G.; Liu, W.; Sun, X. Tuning Electronic Structure of NiFe Layered Double Hydroxides with Vanadium Doping toward High Efficient Electrocatalytic Water Oxidation. *Adv. Energy Mater.* **2018**, *8*, 1703341.

(25) Zhu, Y. P.; Ma, T. Y.; Jaroniec, M.; Qiao, S. Z. Cover Picture: Self-Templating Synthesis of Hollow Co_3O_4 Microtube Arrays for Highly Efficient Water Electrolysis. *Angew. Chem., Int. Ed.* **2017**, *56*, 1161-1161.

(26) Kong, X.; Zhang, C.; Hwang, S. Y.; Chen, Q.; Peng, Z. Free-Standing Holey $Ni(OH)_2$ Nanosheets with Enhanced Activity for Water Oxidation. *Small* **2017**, *13*, 1700334.

(27) Yan, H.; Xie, Y.; Wu, A.; Cai, Z.; Wang, L.; Tian, C.; Zhang, X.; Fu, H. Anion-Modulated HER and OER Activities of 3D Ni–V-Based Interstitial Compound Heterojunctions for High-Efficiency and Stable Overall Water Splitting. *Adv. Mater.* **2019**, *31*, 1901174.

(28) Huang, J.; Sun, Y.; Zhang, Y.; Zou, G.; Yan, C.; Cong, S.; Lei, T.; Dai, X.; Guo, J.; Lu, R.; Li, Y.; Xiong, J. A New Member of Electrocatalysts Based on Nickel Metaphosphate Nanocrystals for Efficient Water Oxidation. *Adv. Mater.* **2018**, *30*, 1705045.

(29) Zhang, B.; Zheng, X.; Voznyy, O.; Comin, R.; Bajdich, M.; García-Melchor, M.; Han, L.; Xu, J.; Liu, M.; Zheng, L.; García de Arquer, F. P.; Dinh, C. T.; Fan, F.; Yuan, M.; Yassitepe, E.; Chen, N.; Regier, T.; Liu, P.; Li, Y.; De Luna, P.; Janmohamed, A.; Xin, H. L.; Yang, H.; Vojvodic, A.; Sargent, E. H. Homogeneously Dispersed Multimetal Oxygen-Evolving Catalysts. *Science* **2016**, *352*, 333.

(30) Yang, Y.; Zhang, K.; Lin, H.; Li, X.; Chan, H. C.; Yang, L.; Gao, Q. MoS_2–Ni_3S_2 Heteronanorods as Efficient and Stable Bifunctional Electrocatalysts for Overall Water Splitting. *ACS Catal.* **2017**, *7*, 2357-2366.

(31) Yan, X.; Li, K.; Lyu, L.; Song, F.; He, J.; Niu, D.; Liu, L.; Hu, X.; Chen, X. From Water Oxidation to Reduction: Transformation from $Ni_xCo_{3-x}O_4$ Nanowires to NiCo/$NiCoO_x$ Heterostructures. *ACS Appl. Mater. Interfaces* **2016**, *8*, 3208-3214.

(32) Jin, Y.; Wang, H.; Li, J.; Yue, X.; Han, Y.; Shen, P. K.; Cui, Y. Porous MoO_2 Nanosheets as Non-noble Bifunctional Electrocatalysts for Overall Water Splitting. *Adv. Mater.* **2016**, *28*, 3785-3790.

(33) Tang, C.; Cheng, N.; Pu, Z.; Xing, W.; Sun, X. NiSe Nanowire Film Supported on Nickel Foam: An Efficient and Stable 3D Bifunctional Electrode for Full Water Splitting. *Angew. Chem., Int. Ed.* **2015**, *54*, 9351-9355.

(34) Jiang, N.; You, B.; Sheng, M.; Sun, Y. Electrodeposited Cobalt-Phosphorous-Derived Films as Competent Bifunctional Catalysts for Overall Water Splitting. *Angew. Chem., Int. Ed.* **2015**, *54*, 6251-6254.

(35) Zheng, J.; Zhou, W.; Liu, T.; Liu, S.; Wang, C.; Guo, L. Homologous NiO//Ni_2P Nanoarrays Grown on Nickel Foams: A Well Matched Electrode Pair with High Stability in Overall Water Splitting. *Nanoscale* **2017**, *9*, 4409-4418.

(36) Jia, Y.; Zhang, L.; Gao, G.; Chen, H.; Wang, B.; Zhou, J.; Soo, M. T.; Hong, M.; Yan, X.; Qian, G.; Zou, J.; Du, A.; Yao, X. A Heterostructure Coupling of Exfoliated Ni–Fe Hydroxide Nanosheet and Defective Graphene as a Bifunctional Electrocatalyst for Overall Water Splitting. *Adv. Mater.* **2017**, *29*, 1700017.

(37) Li, Y.; Zhang, H.; Jiang, M.; Zhang, Q.; He, P.; Sun, X. 3D Self-Supported Fe-Doped Ni_2P Nanosheet Arrays as Bifunctional Catalysts for Overall Water Splitting. *Adv. Funct. Mater.* **2017**, *27*, 1702513.

(38) Gao, X.; Zhang, H.; Li, Q.; Yu, X.; Hong, Z.; Zhang, X.; Liang, C.; Lin, Z. Hierarchical $NiCo_2O_4$ Hollow Microcuboids as Bifunctional Electrocatalysts for Overall Water-Splitting. *Angew. Chem., Int. Ed.* **2016**, *55*, 6290-6294.

(39) Feng, L.-L.; Yu, G.; Wu, Y.; Li, G.-D.; Li, H.; Sun, Y.; Asefa, T.; Chen, W.; Zou, X. High-Index Faceted Ni_3S_2 Nanosheet Arrays as Highly Active and Ultrastable Electrocatalysts for Water Splitting. *J. Am. Chem. Soc.* **2015**, *137*, 14023-14026.

(40) Gong, M.; Zhou, W.; Tsai, M.-C.; Zhou, J.; Guan, M.; Lin, M.-C.; Zhang, B.; Hu, Y.; Wang, D.-Y.; Yang, J.; Pennycook, S. J.; Hwang, B.-J.; Dai, H. Nanoscale Nickel Oxide/nickel Heterostructures for Active Hydrogen Evolution Electrocatalysis. *Nat. Commun.* **2014**, *5*, 4695.

(41) Boppella, R.; Park, J.; Lee, H.; Jang, G.; Moon, J. Hierarchically Structured Bifunctional Electrocatalysts of Stacked Core–Shell $CoS_{1-x}P_x$ Heterostructure Nanosheets for Overall Water Splitting. *Small Methods* **2020**, *4*, 2000043.

(42) Liu, W.; Yu, L.; Yin, R.; Xu, X.; Feng, J.; Jiang, X.; Zheng, D.; Gao, X.; Gao, X.; Que, W.; Ruan, P.; Wu, F.; Shi, W.; Cao, X. Non-3d Metal Modulation of a 2D Ni–Co Heterostructure Array as Multifunctional Electrocatalyst for Portable Overall Water Splitting. *Small* **2020**, *16*, 1906775.
